# Pericardial delta like non‐canonical NOTCH ligand 1 (Dlk1) augments fibrosis in the heart through epithelial to mesenchymal transition

**DOI:** 10.1002/ctm2.1565

**Published:** 2024-02-08

**Authors:** Charlotte Harken Jensen, Rikke Helin Johnsen, Tilde Eskildsen, Christina Baun, Ditte Gry Ellman, Shu Fang, Sara Thornby Bak, Svend Hvidsten, Lars Allan Larsen, Ann Mari Rosager, Lars Peter Riber, Mikael Schneider, Jo De Mey, Mads Thomassen, Mark Burton, Shizuka Uchida, Jorge Laborda, Ditte Caroline Andersen

**Affiliations:** ^1^ Andersen Group, Department of Clinical Biochemistry Odense University Hospital Odense Denmark; ^2^ Clinical Institute, University of Southern Denmark Odense Denmark; ^3^ Department of Cardiovascular and Renal Research Institute of Molecular Medicine, University of Southern Denmark Odense Denmark; ^4^ Department of Nuclear Medicine Odense University Hospital Odense Denmark; ^5^ Department of Cellular and Molecular Medicine University of Copenhagen Copenhagen Denmark; ^6^ Department of Clinical Pathology Sydvestjysk Hospital Esbjerg Denmark; ^7^ Department of Cardiothoracic and Vascular Surgery Odense University Hospital Odense Denmark; ^8^ Department of Clinical Genetics Odense University Hospital Odense Denmark; ^9^ Center for RNA Medicine Department of Clinical Medicine Aalborg University Copenhagen Denmark; ^10^ Department of Inorganic and Organic Chemistry and Biochemistry University of Castilla‐La Mancha Medical School Albacete Spain

**Keywords:** cardiac fibrosis, Delta like non‐canonical NOTCH ligand 1 (Dlk1), epicardium‐derived cells (EPDCs), epithelial to mesenchymal transition, myocardial infarction, myocardial remodelling

## Abstract

**Background:**

Heart failure due to myocardial infarction (MI) involves fibrosis driven by epicardium‐derived cells (EPDCs) and cardiac fibroblasts, but strategies to inhibit and provide cardio‐protection remains poor. The imprinted gene, non‐canonical NOTCH ligand 1 (Dlk1), has previously been shown to mediate fibrosis in the skin, lung and liver, but very little is known on its effect in the heart.

**Methods:**

Herein, human pericardial fluid/plasma and tissue biopsies were assessed for DLK1, whereas the spatiotemporal expression of Dlk1 was determined in mouse hearts. The Dlk1 heart phenotype in normal and MI hearts was assessed in transgenic mice either lacking or overexpressing Dlk1. Finally, in/ex vivo cell studies provided knowledge on the molecular mechanism.

**Results:**

Dlk1 was demonstrated in non‐myocytes of the developing human myocardium but exhibited a restricted pericardial expression in adulthood. Soluble DLK1 was twofold higher in pericardial fluid (median 45.7 [34.7 (IQR)) μg/L] from cardiovascular patients (*n* = 127) than in plasma (median 26.1 μg/L [11.1 (IQR)]. The spatial and temporal expression pattern of *Dlk1* was recapitulated in mouse and rat hearts. Similar to humans lacking Dlk1, adult *Dlk1^−/−^* mice exhibited a relatively mild developmental, although consistent cardiac phenotype with some abnormalities in heart size, shape, thorax orientation and non‐myocyte number, but were functionally normal. However, after MI, scar size was substantially reduced in *Dlk1^−/−^* hearts as compared with *Dlk1^+/+^* littermates. In line, high levels of Dlk1 in transgenic mice *Dlk1^fl/fl^xWT1^GFPCre^* and *Dlk1^fl/fl^* xαMHC^Cre/+Tam^ increased scar size following MI. Further mechanistic and cellular insight demonstrated that pericardial Dlk1 mediates cardiac fibrosis through epithelial to mesenchymal transition (EMT) of the EPDC lineage by maintaining Integrin β8 (*Itgb8*), a major activator of transforming growth factor β and EMT.

**Conclusions:**

Our results suggest that pericardial *Dlk1* embraces a, so far, unnoticed role in the heart augmenting cardiac fibrosis through EMT. Monitoring DLK1 levels as well as targeting pericardial DLK1 may thus offer new venues for cardio‐protection.

## Graphical abstract

Cardiac fibrosis after myocardial infarction (MI) eventually leads to heart failure.

The fibrotic response is mediated by pericardial cells undergoing epithelial to mesenchymal transition (EMT).

We identified Delta like non‐canonical Notch ligand‐1 (*Dlk1*) in the pericardium and its fluid, where it promotes EMT and infarct size.

Targeting Dlk1 thus represents a new strategy for reducing infarct size in MI patients.

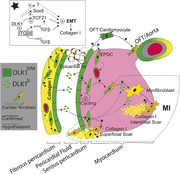

## 1 INTRODUCTION

Myocardial infarction (MI; commonly known as heart attack) is caused by the reduced blood flow to the heart, resulting in apoptosis and necrosis. It includes formation of a fibrotic scar that quickly remodels the infarct zone and prevents cardiac rupture. However, excessive fibrosis in the border and remote zones of the infarcted heart stiffens the heart muscle and reduces cardiac function, eventually leading to heart failure, a major cause of death worldwide. The acute remodelling process after MI is characterised by apoptosis and necrosis, while the subacute process referred to as infarct expansion, including inflammation, fibroblast proliferation and matrix degradation. A final phase of scar maturation then follows with fibroblasts differentiating into myofibroblasts that secrete substantial amounts of extracellular matrix (ECM) components, including collagen.^1^, ^2^, ^3^ The pericardial sac surrounding the heart is a two‐layered structure, where the outer parietal pericardium is highly fibrous and the inner layer (also referred to as the epicardium) consists of mesothelial cell layers forming a pericardial fluid (PF)‐filled space. Detailed fate mapping studies reveals that MI‐remodelling cardiac fibroblasts are derived from the epicardium, where epicardium‐derived cells (EPDCs) proliferate and then undergo epithelial to mesenchymal transition (EMT), differentiating into other cell types (e.g., smooth muscle and cardiac fibroblasts) that migrate and colonise to the myocardium, or stay as EPDCs in close proximity to the epicardium.^4^ Upon MI, interstitial cardiac fibroblasts (iCFs) and EPDCs may undergo EMT, forming collagen I‐secreting myofibroblasts that aggravate cardiac fibrosis. Signalling pathways [e.g., transforming growth factor β (TGFβ), Wnt/β‐catenin, platelet‐derived growth factor (PDGF)] have been shown to mediate both EMT and migration of EPDCs during heart development, and these signalling pathways seem to be reactivated after MI.^1^ Moreover, the peri‐/epicardium itself appears to function as a paracrine organ involved in this fibrotic response after MI,^1^ but very little is known about the process. Molecules in the peri‐/epicardium that facilitate EMT and cardiac fibrosis are thus of considerable interest to identify new therapeutic targets for inhibition of cardiac fibrosis leading to heart failure.

Delta like non‐canonical NOTCH ligand 1 protein [formerly known as Delta‐like 1 homologue (Dlk1)] is associated with several aspects of mammalian development, regeneration and disease. This gene is a part of an imprinted gene network that controls tissue growth.^5^, ^6^, ^7^ *Dlk1* is a maternally imprinted gene (located on mouse chr. 12 and human chr. 14) encoding for an EGF‐like membrane protein, which is similar in structure to the Delta/Notch family of proteins, except for the lack of a DSL domain.^8^ A soluble form of DLK1 is generated by ectodomain cleavage, and both the membrane tethered, and the soluble form seem active.^9^, ^10^, ^11^ The exact function and underlying mechanism of DLK1 remains unclear, although several interaction partners, including NOTCH1 and DLK11 itself, have been suggested.^12^, ^13^, ^14^, ^15^, ^16^ While the function of *Dlk1* in skeletal muscle development and regeneration is known,^7^ current knowledge about the role of *Dlk1* in the heart is very limited, despite its expression in pig, goat and murine hearts.^17^, ^18^, ^19^, ^20^, ^21^, ^22^ Although the expression of *Dlk1* correlates to the regulation of NOTCH signalling and congenital cardiac defects,^19^, ^21^ the defined function of *Dlk1* in the heart remains to be unknown.

Here, we show that *DLK1* is highly expressed in the developing heart and restricted to the pericardium in the adult stage of both mouse and human. *Dlk1* knockout mice display slightly abnormally developed but functionally normal hearts. Using tissue specific gene knockout approach, we find that the scar size is increased in *Dlk1^fl/fl^xWT1^GFPCre^* and *Dlk1^fl/fl^* xαMHC^Cre/+Tam^ mice, whereas Dlk1 knockouts exhibit decreased scar size. Mechanistically, Itgb8 is reduced in *Dlk1* ^−/−^ mice, accompanied by reduction of EMT. Taken together, our study suggests that pericardial *Dlk1* affects cardiac fibrosis through either EMT of interstitial fibroblasts or EPDCs.

## 2 MATERIALS AND METHODS

An extended methods section is provided in supporting information present online.

### 2.1 Human samples

For DLK1 analysis in the myocardium, we collected: (i) post‐mortem heart ventricle tissues from 22 individuals, (ii) human foetal heart tissue [*n* = 26; 42−280 days post fertilisation (dpf)] and (iii) patients undergoing valve replacement surgery. The age of the 22 individuals ranged from 17 to 98 (median: normal (51.5), acute MI (65.5), chronic MI (65), hypertrophic (60.5)) and included 13 males and nine females. The cause of death in the different groups included: normal (car, train, work, and drowning accidents), acute MI (MI), chronic MI (lung diseases, stomach complications and sequelae from previous infarct), hypertrophic (lung diseases/injury, pancreatic cancer, aorta dissection). For human foetal heart tissue, GA ranged from 91 to 280 days dpf, and included 13 male and 13 female specimens, where heart tissue showing haemolysis due to intrauterine death was excluded from the analysis. Foetuses were either normal (*n* = 9) or exhibited known/unknown genetic abnormalities (*n* = 9; Trisomy, Turner and others where *n* = 3 exhibited heart defects), intestinal malrotations/hernia (*n* = 3), brain abnormalities (*n* = 3) or placenta defects (*n* = 3). For pericardial samples, we obtained plasma and PFs (*n* = 127), as well as parietal pericardial tissue biopsies (*n* = 12) from patients elected for coronary artery bypass grafting (CABG) or cardiac valve replacement surgeries^23^, ^24^ (Figure S1). We obtained informed consent from enrolled subjects in agreement with ethical legislation. The three human protocols of the study were in accordance with the Declaration of Helsinki and were approved by the Medical Ethical Committee of the Region of Southern Denmark (Protocols: #S‐20120065, #S‐20180056, #S‐20100044).

### 2.2 Animal experiments

C57BL/6J mice, *Dlk1^−/−^*,^25^, ^26^ αMHC‐MerCreMer,^27^ WT1^GFPCre^, and *Dlk1^fl/fl^* Jl mice (generated herein) were crossbred as described in the supporting information present online. Sprague–Dawley rats used for in vitro and in vivo analysis are described in detail in the supporting information present online. MI was introduced by permanent ligation of the of the left anterior descending (LAD) coronary artery. The heart function was assessed by F‐18‐fluorodeoxyglucose positron emission tomography (18FDG‐PET). ^18^FDG‐PET images were analysed by a blinded physicist with high experience in using the QGS software (Cedars‐Sinai Medical Center, Los Angeles, CA, USA). Pericardial sac lesion (PSL) was done similarly to LAD ligation, although the myocardium was left undisturbed. Three‐lead electrocardiograms (ECG) were recorded using BioVet CT1 system software (M2M Imaging), while CT scanning was used to quantify coronary artery length. All animal experiments were approved by the Danish Council for Supervision with Experimental Animals (#2016‐15‐0201‐00941).

### 2.3 Histology, flow cytometry, mRNA microarray, qRT‐PCR and ELISAs

For histology, dissected hearts or biopsies were embedded or snap‐frozen, followed by slicing throughout in steps to reveal all parts of the hearts. Sections were stained and evaluated by microscopy. Flow cytometry was performed as previously described^7^, ^28^, ^29^ on fixed cardiac cells (either EPDCs, cardiac fibroblasts or all cardiac cells). Genome wide transcriptomic profiling was performed using Affymetrix^®^ GeneChips (Mouse Genome 430 2.0 Array) that were run using the GeneChip Scanner 3000 (Affymetrix, Santa Clara, CA, USA). For qRT‐PCR assay, total RNA was extracted from cells or tissues by TRIzol. cDNA synthesis and qRT‐PCR were performed as previously described^7^ and normalised by the qBase Plus platform.^30^ DLK1 ELISAs of soluble DLK1 in PF, blood or medium was quantified using inhouse assays.^23^, ^31^, ^32^

### 2.4 Isolation and culturing of cells

Neonatal EPDCs were isolated and cultured as previously described.^29^ The transfection of siRNAs [Itgb8 (ID s115991) and Scramble (Ambion, Thermo Fisher Scientific)] was performed as previously described.^11^ Adult cardiac fibroblasts were isolated by the heart dissociation kit (Miltenyi Biotec) and cultured upon transfection with plasmids harbouring full‐length *Dlk1* (DLK1FL‐pLHCX‐HA), soluble *Dlk1* (DLK1E‐pLHCX‐HA) or an empty control plasmid (pLHCX‐HA). Adult cardiac cells for flow cytometry analysis were isolated using the Langendorff adapted EasyCell isolation system.

### 2.5 Statistical analyses

All data are presented in the figures, and each analysis consisted of at least three independent experiments analysed by appropriate statistical tests depending on normality (D'Agostino & Pearson) of the data. Individual tests are indicated in the figure legends and include: two‐way ANOVA (ordinary or repeated) followed by appropriate posthoc analysis as indicated, including unpaired *t*‐test, Wilcoxon matched‐pairs signed rank test, simple linear regression, Mann–Whitney test, Kruskal–Wallis test with two‐stage linear step‐up procedure of Benjamini, Krieger and Yekutieli, and Chi‐square test.

### 2.6 Data sharing

The data that support the findings of this study are available from the corresponding author upon reasonable request.

## 3 RESULTS

### 3.1 DLK1 is secreted from the human peri‐/epicardium into the PF

**FIGURE 1:**
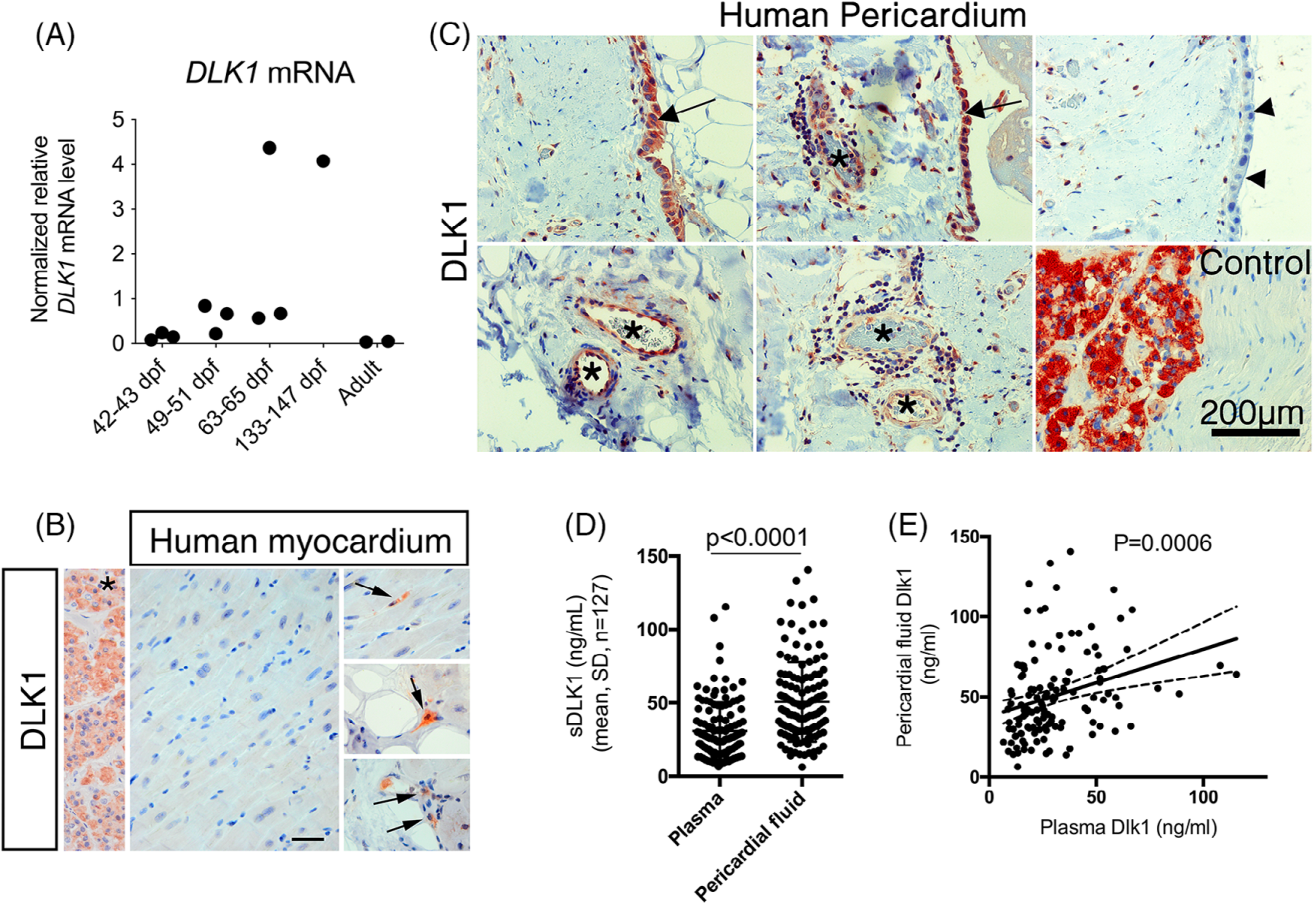
Delta like non‐canonical notch ligand 1 (Dlk1) in the human heart is restricted to cells in the pericardium and soluble sDLK1 is secreted into the pericardial fluid. (A) Relative quantitative real time PCR of *DLK1* in human myocardial specimens during development (days post‐fertilisatioin, dpf) and adulthood (*n* = 1–3). *B2M*, *ATP6A* and *COX4A* were used for normalisation. (B) DLK1 immunohistochemistry of normal human myocardium (*n* = 3) and *pituitary gland as positive control. Arrows point at discrete non‐muscle cells expressing DLK1. (C) DLK1 immunohistochemistry of human pericardium (*n* = 12) and positive control tissue (pituitary gland). Arrows and arrowheads point at DLK1+ and DLK1− mesothelial cells, respectively, while * marks vasculature with a DLK1+ endothelium. (D and E) Soluble DLK1 was measured in corresponding plasma and pericardial fluid samples from patients (*n* = 127) undergoing heart valve or coronary artery surgery. Wilcoxon matched‐pairs signed rank test (D) and simple linear regression (E) were used to test statistical significance. See Figure S1 for further details.

Earlier studies demonstrated high expression of *Dlk1* in the outflow tract (OFT) endocardium, epicardium, atrial septum and mesenchyme of the cushions in the embryonic (E12.5–E13.5) mouse heart.^19^, ^21^ To further examine the expression pattern of *DLK1* in humans, we analysed normal human embryonic and adult hearts (Figure 1A). The results shows that *DLK1* mRNA levels increased during human heart development and then declined to become nearly abolished in normal adult ventricles. This was confirmed by immunohistochemistry showing that DLK1 protein localises to many non‐cardiomyocytes in the developing human heart (Figures S1A and B), whereas DLK1 is rarely detected in the ventricle of normal adults (Figures 1B and S1A and B), although occasional non‐myocyte DLK1+ cells were observed in the vicinity of mainly vessels (Figure 1B). Moreover, DLK1+ cells were nearly absent in the myocardium from patients suffering from acute MI (*n* = 7 patients), chronic MI (*n* = 6 patients) or cardiac hypertrophy (*n* = 8 patients) (Figures S1A and B). On the contrary, in pericardial tissue specimens from another cohort of patients undergoing cardiothoracic surgery for residual cardiovascular disease (i.e., coronary artery disease or cardiac valve failure), we found substantial and consistent DLK1 expression (*n* = 12 patients) in the pericardium, where DLK1 localised to discrete stretches of mesothelial cells (Figure 1C). Furthermore, vascular structures, including endothelial cells, within this compartment as well some stromal cells interspersed within the collagen matrix, exhibiting a distinctive DLK1 expression pattern (Figure 1C).

As many of the above identified DLK1+ structures are faced towards the lumen of the pericardial space, we hypothesised that the soluble form of DLK1 (sDLK1) was secreted into and present in PF. To test this hypothesis, we collected PF and venous plasma samples from a cohort of 127 patients (patient characteristics summarised in Figure S1C) undergoing valve or CABG and quantified for sDLK1 amounts therein. The results exposed no gender and no disease aetiology (valve or coronary artery disease) related differences in sDLK1 levels for either plasma or PF (Figures S1D–F). In addition, the sDLK1 levels did not correlate to classical risk factors (continuous data), possibly due to the extensive drug treatment received by the patients (Figure S1C). Thus, as no immediate correlations to obvious stratifications were present, data were pooled for all 127 patients. The median sDLK1 concentration in venous plasma was 26.1 μg/L (11.1 (IQR)) of these cardiovascular disease patients, which is within the normal range.^31^ In contrast, the sDLK1 concentration in the PF compartment [median 45.7 (34.7 (IQR)) μg/L] was significantly (*p* < .0001) more abundant (Figure 1D). The mean sDLK1 ratio ([PF]/[*P*]) was 2.041 ± 1.3 (SD) fold higher in PF than in venous plasma. In addition, sDLK1 levels in the two compartments showed a strong linear correlation (*p* = .0006; Figure 1E). Moreover, we observed that the sDLK1 ratio ([PF]/[*P*]) correlated with that of metabolic syndrome markers associated with cardiovascular disease,^23^, ^24^ including soluble Tweak (*r* = 0.3175, *p* < .0001), Adiponectin (*r* = 0.4689, *p* = .002), A‐FABP (*r* = 0.3332, *p* < .0001), lipocalin 2 (*r* = 0.3175, *p* = .0166) and hs‐CRP (*r* = −0.2320, *p* = .0009), but not total protein, leptin, proteinase 3, BNP and elastase (data not shown). These data strongly suggest that in humans, *DLK1* is expressed in the peri‐/epicardial cell lineage during development, whereas in the adult heart it becomes restricted to the pericardium where it is secreted into the PF.

### 3.2 DLK1 is expressed in the EPDC lineage during mouse heart development

**FIGURE 2:**
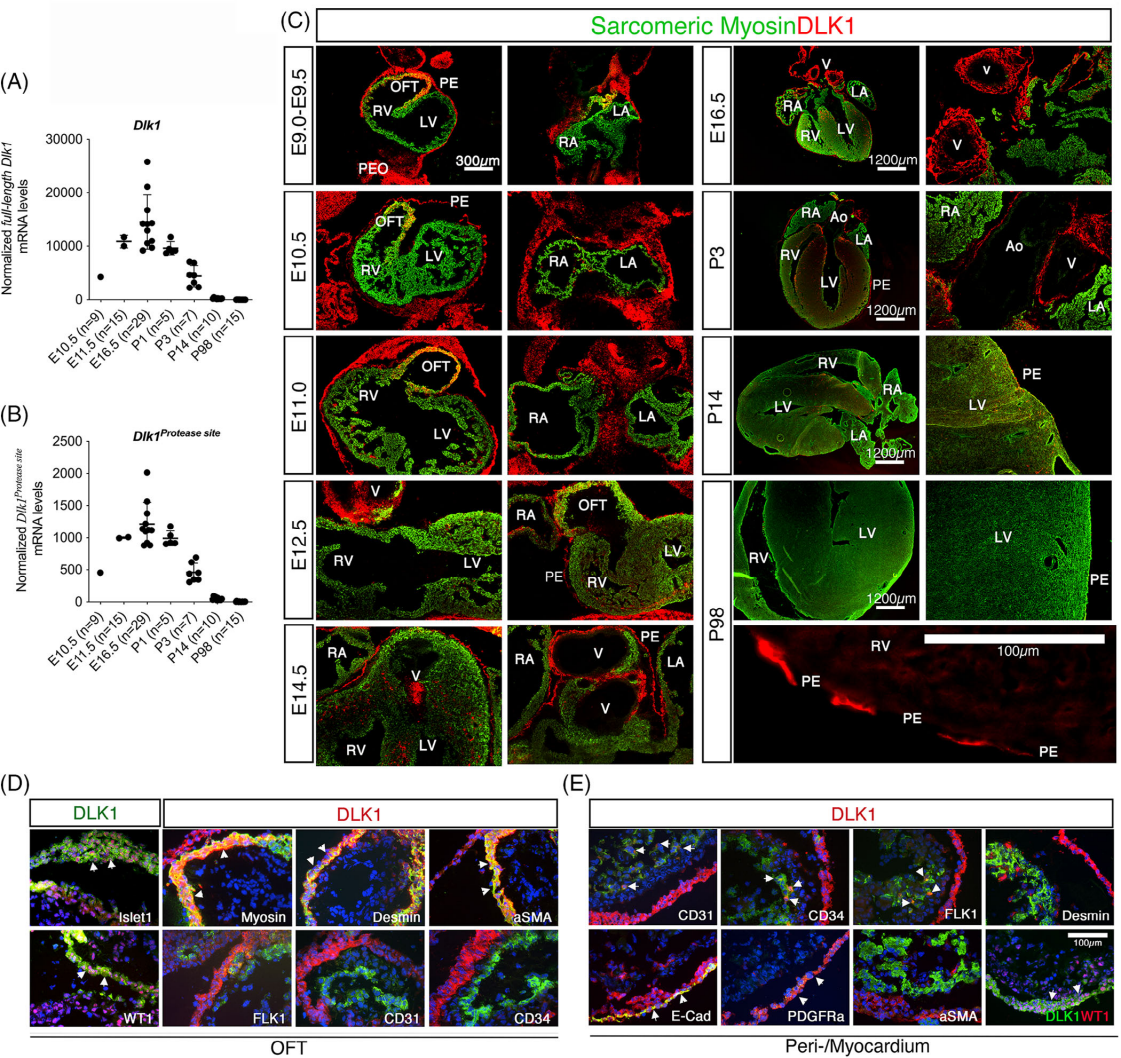
Dlk1 localises to the secondary heart field during mouse cardiac development. (A and B) Relative mRNA expression levels of full‐length and *Dlk1^Protease site^* mRNA (Dlk1 variants that comprise the protease site resulting in soluble Dlk1 isoforms) during mouse heart development. Number of animals for each sample is depicted on graphs. Raw data were normalised against *Gapdh* and *beta‐actin* (qbase+: M = 0.542; CV = 0.187). (C) DLK1 (red) and sarcomeric Myosin (green) immunofluorescence of hearts during development and adulthood (*n* = 2–4 animals deriving from different litters for each timepoint were analysed, representative pictures shown. See Figure S2 for further details. (D and E) DLK1 is co‐expressed with markers of epicardial‐derived progenitor cells (EPDCs) at E10.5 in EPDCs and descendants residing in (D) the outflow tract (OFT) and within (E) the peri‐/myocardium as visualised by immunofluorescence (*n* = 3 animals deriving from different litters were analysed, representative pictures shown). DAPI (blue) was used for staining nuclei.

To further explore the temporal and spatial expression of *Dlk1* mRNA and protein in the heart, we profiled C57BL/6 mice from E9.0 until adulthood. The expression of full‐length *Dlk1* increased during mouse heart embryogenesis, decreased in neonates and abolished in adult mouse hearts (Figure 2A). The same was true for *Dlk1^Protease site^* mRNA encoding the soluble variant of Dlk1 (Figure 2B). These expression patterns reflect what was observed in the human heart (Figure 1A). The careful inspection of E9.0–9.5 hearts revealed that DLK1 protein localised to the parietal pericardium, the proepicardial organ, some interstitial non‐myocytes (myosin negative) in the right ventricle and numerous cells in the OFT (Figures 2C and S2).

In the E10.5–E11 hearts, these DLK1 expression patterns were extended to the visceral pericardium, the left ventricle and a few cells lining myocytes throughout the atria (Figures 2C and S2). At E10.5, the OFT residing DLK1^+^ cells co‐expressed the transcription factors and EPDC markers, Islet1 (ISL LIM Homeobox 1) and Wt1 (Wilms tumour protein) (Figure 2D). Furthermore, the expression of DLK1 protein coincides with those of structural cardiomyogenic proteins, such as Myosin, alpha Smooth Muscle Actin (aSMA) and Desmin (Figure 2D), which are known to be expressed in the transient OFT structure that forms the base of the aorta.^33^ Within the ventricle wall, DLK1 was expressed in the developing capillary vasculature (which are CD31^+^/CD34^+^/FLK1^+^), although it did not co‐localise with the cardiomyocyte proteins (Figure 2E). A careful examination of the peri‐/epicardium revealed that DLK1^+^ cells co‐express aSMA^Low^, PDGFRa, E‐Cadherin and WT1 (Figure 2E) but not Islet 1 (data not shown), suggesting that Dlk1 is expressed in EPDCs and their descendants. Although the epicardium and pericardium are very thin in the mouse, DLK1 clearly localised to both epicardium and pericardium based on E‐Cadherin expression (Figure 2E). From E12.5 until postnatal day three (P3), DLK1 expression was high in the mesenchyme surrounding the larger vessels, but it was also markedly expressed in the parietal‐/visceral pericardium, small interstitial cells in the ventricles, and small cells lining the myocytes in the atria (Figures 2C and S2). The vascular‐associated DLK1 expression was substantial at E16.5 when DLK1 seemed to mark a large fraction of the developing CD31^+^/CD34^+^/FLK1^+^ capillary vasculature within the myocardium but not in large vessels, where it was expressed in fibroblasts in the adventitia (Figure S3A).

However, this was not the case at postnatal day 3 (P3), where DLK1, besides the expression in the peri‐/epicardium, was merely expressed in interstitial long‐stretched cells with a fibroblast‐like CD34^+^/DDR2^+^/Laminin^+^/Vimentin^+^ phenotype (Figure S3B). At P14, only a few DLK1^+^ cells were scattered around in the ventricles and atria, as well as in some mesenchymal structures, such as papillary muscles, AV‐junction, AV/aortic valves and septum. Also at this stage, large vessels were DLK1 negative, but surrounding adventitia was DLK1 positive. The epicardium and, to a lesser extent, the pericardium remained DLK1 positive (Figures 2C and S2). However, at P98 (referring to adulthood), DLK1 was nearly absent throughout the heart, except for a few non‐myocytic cells and some long‐stretched DLK1^+^ cells lining the pericardium, the interior of the aorta and in the surrounding mesenchyme (Figures 2C and S2). Similarly, we found that DLK1 expression patterns in adult rats parallels the mouse and human data with a few scattered DLK1+ cells of non‐myocytic identity existing within the rat myocardium (Figure S4A). Taken together, our human and murine data suggest that a first wave of DLK1^+^ cells in the embryonic heart marks early multipotent progenitors of the EPDC cell lineage, including vascular cells, whereas a second neonatal wave of DLK1^+^ cells primarily comprise cardiac fibroblast descendants. However, in adulthood, DLK1 expression is mainly restricted to peri‐/epicardial cells.

### 3.3 *Dlk1^−/−^* hearts are abnormally developed but appear with an overall modest phenotype

**FIGURE 3:**
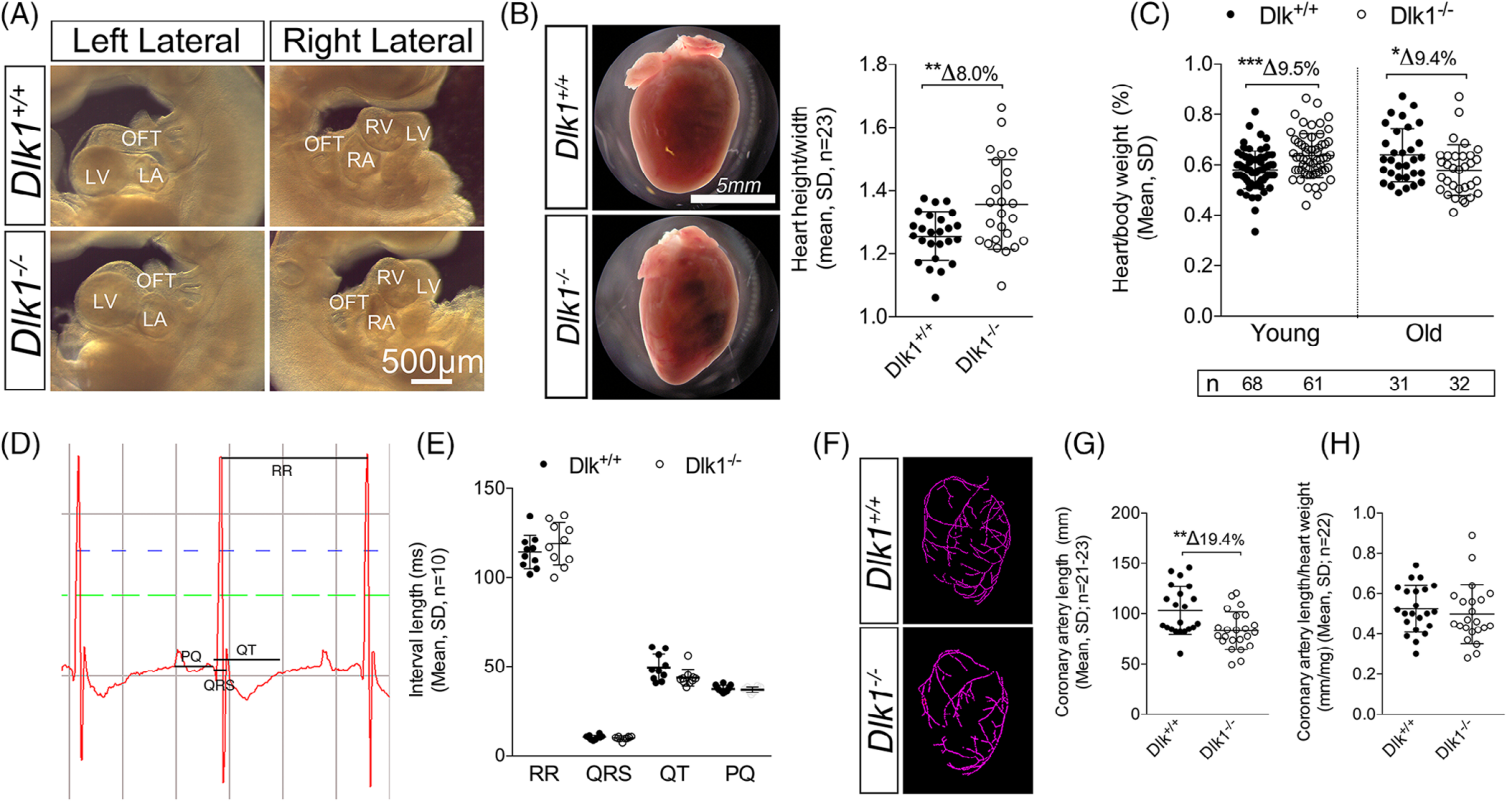
*Dlk1^−/−^* hearts display modest abnormal growth. Stereomicroscopic dark field pictures of (A) E10.5− and (B) adult mouse *Dlk1^+/+^* and *Dlk1^−/−^* hearts. Independent of sex (Figure S5), heart height/width was increased in adult *Dlk1^−/−^* hearts, whereas (C) heart/body weight was increased in young (P1–P3) and decreased in adult‐old (P79–P436) *Dlk1^−/−^* hearts as compared with *Dlk1^+/+^* hearts. (D) Electrocardiography (representative) showed that (E) electrical heart activity was similar in adult *Dlk1^+/+^* and *Dlk1^−/−^* mice independent of sex. (F) Microfil‐injected coronary beds of *Dlk1^+/+^* and *Dlk1^−/−^* hearts were visualised by CT scanning and used for (G) quantifying coronary artery length, which was further (H) normalised to heart weight. For all sets of data, we confirmed sex independency by two‐way ANOVA, and then in the accumulated data sets statistical significance was tested using unpaired *t*‐test (B and G) or Mann–Whitney (C, E and H) depending on data normality (D'Agostino & Pearson). LV, left ventricle; LA, left atrium; OFT, outflow tract; RV, right ventricle; RA, right atrium.

**FIGURE 4:**
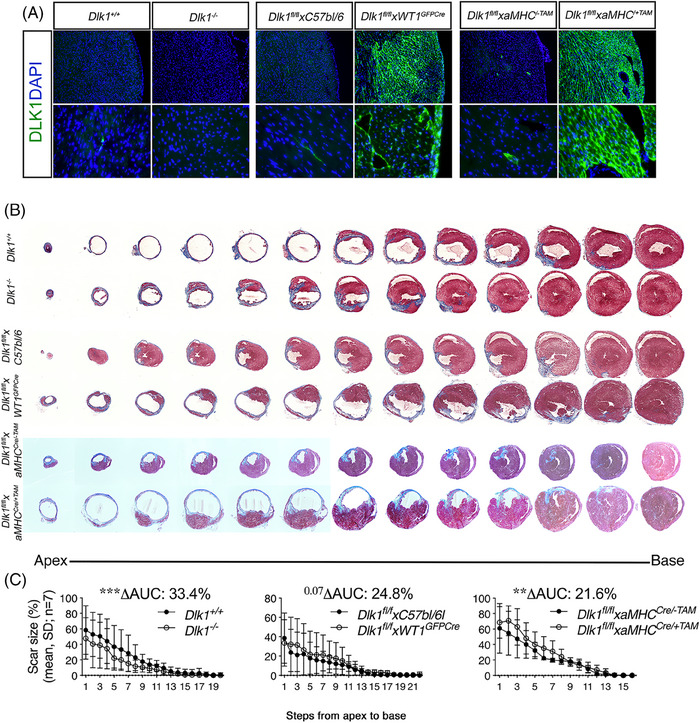
Dlk1 increases scar size following myocardial infarction (MI). (A–C) MI (LAD) or sham surgery was performed in 10‐weeks old female C57bl/6 mice (cardiac stress markers, see Figure S7A). (A) Immunofluorescence images of hearts from three sets of generated transgenic mice: *Dlk1^−/−^* (dlk1 deletion), *Dlk1^fl/fl^xWT1^GFPCre^* (EPDC lineage *Dlk1* overexpression) and *Dlk1^fl/fl^* xαMHC^Cre/+Tam^ (EPDC environment *Dlk1* overexpression) and their corresponding controls. MI was verified at 6 weeks by PET scanning (Figure S8‐S10), and (B) after 8 weeks by scar size measurements using serial Massons’ Trichrome stained sections from apex to base, which was used to (C) quantitate scar size in a 3D‐like manner by calculating the area under the curve of all steps. For statistical testing, we used two‐way ANOVA. Each MI series of transgenic and littermate control was performed by different operators and different blinded analysers, limiting comparisons between sets.

To investigate Dlk1's functional role in the heart, we studied homozygous *Dlk1^−/−^* and wildtype *Dlk1^+/+^* mice.^26^ Surprisingly, *Dlk1^−/−^* mice were fully viable, showed similar litter sizes and Mendelian ratios to their wildtype littermates (Figures S5A and B). However, *Dlk1^−/−^* mice showed growth retardation reflected by reduced body weight (*p* < .0001, *n* = 229 mice, age: P1–436) as previously described.^7^ To further examine whether if there is any difference between *Dlk1^−/−^* and *Dlk1^+/+^* mice, we carefully dissected their embryonic hearts. At E10.5, no major difference was observed in overall heart appearance between *Dlk1^−/−^* and *Dlk1^+/+^* mice (Figure 3A). However, the postnatal/adult *Dlk1^−/−^* heart was shaped differently with an increased heart height to width ratio (Figures 3B and S5C–E). Furthermore, it was oriented almost perpendicular to the midline of the mouse with the apex pointing to the left (Figure S5F). These phenotypes are independent of sex of mice. On average, young (P1–P3) *Dlk1^−/−^* hearts were enlarged by 9.5% (mean; *n* = 61−68 litters), whereas the opposite was observed in adulthood (P79–436) with a 9.4% (mean; *n* = 31−32 mice) reduction in heart/body ratio for *Dlk1^−/−^* animals (Figure 4C).

Despite the above observed differences in the heart size, we did not observe any difference in adult cardiomyocyte size (Figure S5G). Furthermore, besides minor negative deflections in 50% of *Dlk1^−/−^* hearts following the P peak, which was described previously for mice,^34^ we did not observe any major difference between *Dlk1^+/+^* and *Dlk1^−/−^* hearts with respect to conductivity determined by ECG of live adult animals (Figures 3D–E). In addition, microscopic analysis did not reveal any major defects in adult valve formations (data not shown). Regarding vascularity, no apparent difference was seen in overall architecture of the large coronary vessels between *Dlk1^+/+^* and *Dlk1^−/−^* hearts using micro CT scanning (Figure 3F). The total coronary artery length was substantially reduced in *Dlk1^−/−^* hearts (Figure 3G), which correlates well to the lower heart weights observed for *Dlk1^−/−^* animals (Figure 3H).

To further confirm these findings, we dissociated adult heart ventricles and performed flow cytometry analysis, which indicated a 10 percent decrease in non‐myocytes (MYH1^−^) in *Dlk1^−/−^* mouse ventricles as compared with *Dlk1^+/+^* ventricles (Figure S6), but no obvious difference between genotypes in the percentage of ventricular cardiac fibroblasts (aSMA^+^, Vimentin^+^, CD90^+^, PDGFRα^+^) (Figure S6). Taken together, the deletion of *Dlk1* seems to influence heart growth, albeit modestly. This is in accordance with cardiomyopathy being milder in humans with 14q32 deletions (devoid of *Dlk1*) than in partial trisomy of 14q32 exhibiting high Dlk1 expression.^35^, ^36^

### 3.4 *Dlk1* expression exaggerates scar size after MI

Both EPDCs and epicardium‐derived iCFs have been shown as major contributors to myocardial remodelling and scarring after MI.^1^, ^37^ Since our data show that Dlk1 is restricted to cells in the adult peri‐/epicardium (Figures 1 and 2), we next aimed to elucidate whether *Dlk1* plays a role in MI‐induced myocardial remodelling (Figure S7A). Although DLK1 expression itself did not increase neither systemically nor locally in the infarcted heart by permanent ligation of coronary artery, its expression increased upon pericardial lesion (Figures S7B–E). Similarly, Dlk1 was also not induced in the myocardium by transverse‐aortic‐constriction (Figures S7F and G), that represents another type of heart injury with diffuse fibrosis throughout the left ventricle and which leads to hypertrophy. Although, we cannot exclude that Dlk1 even so is implicated in fibrosis after hypertrophy, our mouse data correlate to those in humans (Figures 1 and S1A and B) and show that Dlk1 is consistently expressed in the

pericardium, and this expression seems increased by pericardial lesion.

Since cardiomyopathy is mild in humans with 14q32 deletions (*Dlk1 absence*), yet severe in partial trisomy of 14q32 with Dlk1 overexpression,^35^, ^36^ we hence speculated whether abnormal high Dlk1 levels present either in the pericardium or the myocardium may impact the scarring process after MI. To address this point, we generated three sets of Dlk1 mice to mimic situations of high and low Dlk1 in the heart: (i) *Dlk1^−/−^* mice (to evaluate the effect of Dlk1 originating within the pericardial compartment in adulthood), (ii) *Dlk1^fl/fl^* × *WT1^GFPCre^* (to assess high levels of Dlk1 specifically in the EPDCs lineage throughout the heart) and (iii) *Dlk1^fl/fl^* × *αMHC^Cre/+Tam^* (to test high levels of Dlk1 present in the environment and vicinity of the EPDC lineage cells) (Figure 4A).

Myocardial function was assessed by Positron‐emission tomography (^18^FDG‐PET) 1 week before and 6 weeks after MI induction (Figures S8–S10). To compensate for baseline differences within genotypes, analysis was performed as repeated measures. Despite an expected and slightly decreased stroke volume in the smaller *Dlk1^−/−^* mouse (Figure S8), overall cardiac function (% ejection fraction) was similar at baseline for all genotypes as compared with controls but decreased upon LAD artery ligation (Figures S8–S10). For *Dlk1^fl/fl^* × *αMHC^Cre/+Tam^* mice, we found a substantial reduction in cardiac function as compared with their controls (Figure S9). In contrast, *Dlk1^fl/fl^* × *WT1^GFPCre^* and *Dlk1^−/−^* mice were comparable to their respective controls 6 weeks after MI (Figures S8 and S10). Interestingly, the wall thinning was clearly apparent already at baseline for both Dlk1 overexpressing animals (Figures S9 and S10), and substantial for *Dlk1^fl/fl^* × *αMHC^Cre/+Tam^* mice after 6 weeks. This suggest that high levels of Dlk1 in the heart by itself confers a disadvantage to the ventricle wall, whereas deleting Dlk1 only resembles a mild cardiac phenotype as already notice above (Figure 3).

To evaluate scar size eight weeks after MI, we comprehensively sliced the hearts from apex to base, and used these serial sections (Figure 4B) to create 3D‐like quantifications of the scar area (Figure 4C). By calculating area under the curve, we found that scar size was decreased significantly by 33.4% in *Dlk1^−/−^* hearts but significantly increased 21.6% in *Dlk1^fl/fl^* × *αMHC^Cre/+Tam^* and tended (*p* = .07) to be increased with 24.8% in *Dlk1^fl/fl^* × *WT1^GFPCre^* hearts as well (Figure 4C). Thus, data from these sets of mice showed that the presence of *Dlk1* after MI in adulthood negatively affects cardiac remodelling.

### 3.5 *Dlk1* promotes EMT of EPDCs through Itgb8

**FIGURE 5:**
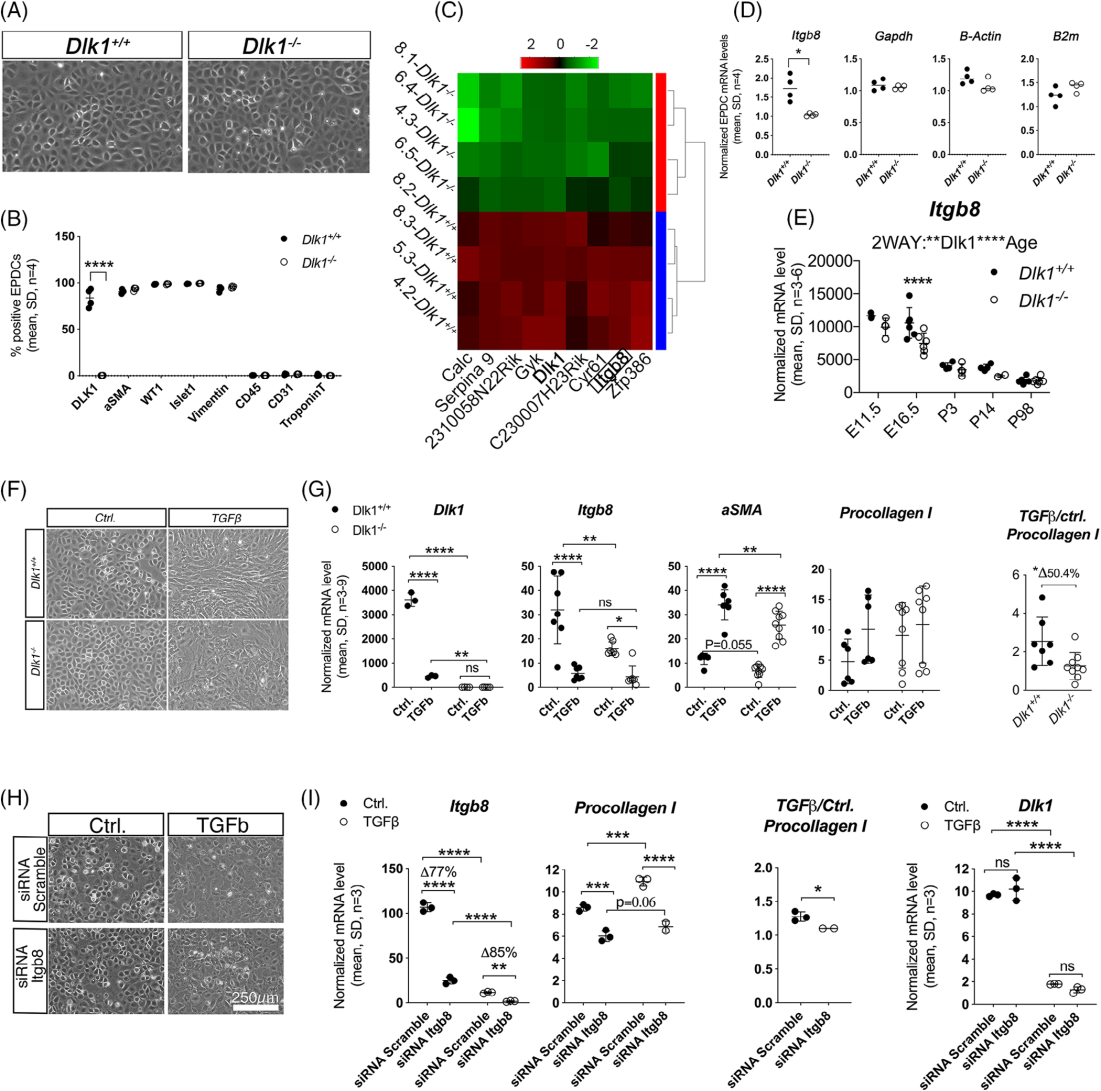
*Dlk1^−/−^* EPDCs exhibit a reduced ability for TGFβ‐mediated EMT. (A) Typical cobblestone morphology of undifferentiated EPDCs obtained from neonatal *Dlk1^+/+^* and *Dlk1^−/−^* hearts (*n* = 4) that (B) except for DLK1, display very similar marker expression and culture purity (CD45, CD31 and TroponinT) as judged by flow cytometry and furthermore, (C) genome wide profiling only identified a limited number of differentially expressed genes. (D and E) Relative quantitative RT‐PCR validated *Itgb8* gene array data in in vitro cultured EPDCs and in *Dlk1^+/+^* and *Dlk1^−/−^* hearts during development. For D, *Gapdh, beta‐actin* and *B2m* were used for normalisation. (F and G) *Dlk1^+/+^* and *Dlk1^−/−^* EPDCs were stimulated with TGFβ or vehicle (control) to undergo fibroblast differentiation, which was verified by (F) a morphology change and (G) qRT‐PCR. Dlk1 and Itgb8 was as expected reduced in *Dlk1^−/−^* EPDCs as compared with *Dlk1^+/+^* EPDCs, which was accompanied by lower expression of the cardiac fibroblast EMT markers aSMA and Procollagen I. (H and I) *Itgb8* knock down by 75−90% in *Dlk1^+/+^* EPDCs was accompanied by a decrease in cardiac fibroblast differentiation (preserved epithelial morphology, low *Procollagen I*), but with no effect on *Dlk1* itself. For (G and I) *Procollagen I* levels were normalised within each experiment to compensate the observed inter‐experiment variation and tested by non‐parametric Mann–Whitney. For all qRT‐PCR data (as exemplified in (D)), normalisation was performed against several stably expressed endogenous controls according to the qBase platform (see *Material and Methods* section). For all other statistical testing, we used two‐way ANOVA/Fisher's LSD (B), paired *t*‐test (D), two‐way ANOVA/Holm‐Sidak's test (E, G and I).

We next aimed to elucidate at the cellular level how *Dlk1* is implicated in EMT of EPDC, a key event in the fibrotic remodelling during heart development and after MI as observed above. As disease and injury to the heart and the pericardium often leads to re‐expression of embryonic/foetal genes,^38^ we chose to use neonatal EPDCs. To this end, we stripped off the mouse peri‐/epicardium (Figure S11A) and established pure in vitro cultures of *Dlk1^+/+^* and *Dlk1^−/−^* neonatal EPDCs (Figures 5A and B). The expression of DLK1 in *Dlk1^+/+^* EPDCs seemed dynamic as the sorted DLK1^−^ cells from *Dlk1^+/+^* EPDCs gave rise to DLK1^+^ EPDCs when evaluated at day 10 in culture (Figures S11B and C). This may explain why only 83.7 ± 9.9% (*n* = 4) of *Dlk1^+/+^* EPDCs were positive for DLK1 at a given time point (Figure 5B). In agreement with previous observations for several cell types,^11^, ^39^ we found an increased proliferation potential of *Dlk1‐*deficient EPDCs in vitro (Figures S11E–G).

To dissect the molecular mechanism, global gene expression profiling of *Dlk1^+/+^* and *Dlk1^−/−^* EPDCs was performed. Using high stringency, it is revealed that only a small number of genes was robustly affected by *Dlk1* (Figure 5C). Among them, Integrin β8 subunit (ITGB8) gene, a recently demonstrated activator of TGFβ,^40^ was substantially decreased in *Dlk1^−/−^* EPDCs (Figure 5D). Overall, *Itgb8* levels were similar in a large range of adult *Dlk1^+/+^* and *Dlk1^−/−^* tissues (Figure S12), thus excluding that the observed *Itgb8* downregulation in *Dlk1^−/−^* EPDCs could be due to an artefact generated on chromosome 12, where both genes are located. Moreover, we found that *Itgb8* levels were reduced in *Dlk1^−/−^* hearts during cardiac development (Figure 5E) with a substantial reduction at E16.5 (Figure 5E) when *Dlk1* levels peak in normal *Dlk1^+/+^* hearts (Figures 2A and B).

Upon stimulation with the major EMT‐inducer TGFβ, EMT was inhibited in *Dlk1^−/−^* EPDCs as compared with *Dlk1^+/+^* EPDCs (Figures 5F and G). Notably, *Dlk1* expression itself vanished in differentiated EPDCs (Figure 5G), suggesting a role mainly occurring before or at early stages of EMT of EPDCs. Interestingly, this spatial DLK1 expression profile was recapitulated in rat EPDCs (Figures S4B and C). Taken together, these data reflect the Dlk1 expression profile seen in vivo (Figures 1 and 2), where migrated and differentiated EPDCs within the myocardium stopped expressing *Dlk1* with further development of the heart.

To evaluate the likelihood of *Itgb8* contributing to EMT of EPDCs, we performed siRNA‐mediated knock down of *Itgb8* in *Dlk1^+/+^* EPDCs (Figures 5H and I) and observed a 30 and 37% reduction in Procollagen1 with no effect on *Dlk1* itself (Figure 5I). Thus, collectively, these data support a mechanism whereby *Dlk1* maintains *Itgb8* levels, enabling or substantiating TGFβ’s activity to negatively affect the fibrotic response after MI.

### 3.6 EMT of EDPCs and iCFs with collagen I production is enhanced by Dlk1

**FIGURE 6:**
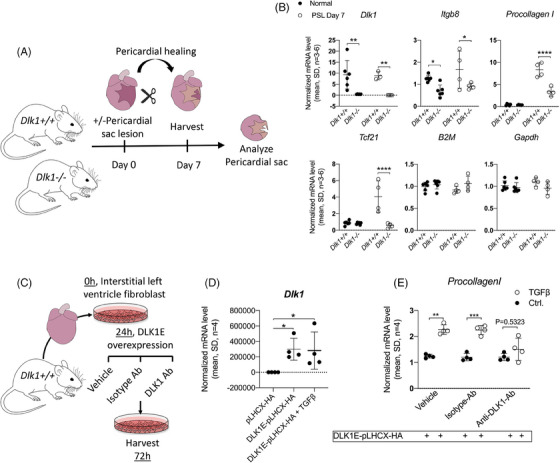
*Dlk1* affects heart fibrosis through increased collagen I expression in the pericardium and in interstitial cardiac fibroblasts. (A) The pericardial sac was dissected from adult *Dlk1^+/+^* and *Dlk1^−/−^* animals (*n* = 4–6) at day 7 after ± pericardial lesion and analysed by (B) qRT‐PCR for *Dlk1*, *Itgb8*, *Procollagen I* and *Tcf21* expression. Expression was normalised against *B2M* and *Gapdh* (M:0.34 and CV:0.12) using qBase+ (C–E) Interstitial cardiac ventricle fibroblasts were isolated from adult mouse hearts, cultured and transfected with the extracellular cleavable Dlk1 gene (DLK1E‐pLHCX‐HA) or an empty vector (pLHCX‐HA). Dlk1 expressing cells were stimulated with ±TGFβ and treated with vehicle, isotype‐antibody (Ab) or anti‐DLK1‐Ab before qRT‐PCR for *Procollagen I* expression. For all qRT‐PCR data (as exemplified in (B)), normalisation was performed against several stably expressed endogenous controls according to the qBase platform (see *Materials and Methods* section). For statistical testing, we used two‐way ANOVA/Holm‐Sidak's test (B) and non‐parametric Kruskal–Wallis/two‐stage linear step‐up procedure of Benjamini, Krieger and Yekutieli (D and E).

Given that the fibrotic response following MI may be mediated both by EMT of EPDCs in the pericardium and/or by EMT of interstitial fibroblasts (iCFs),^1^, ^37^ we then asked whether reactivation of the pericardium alone may trigger a *Dlk1*‐dependent EMT response in vivo within the pericardium. To test this point, we performed PSLs in *Dlk1^+/+^* and *Dlk1^−/−^* animals and analysed the dissected pericardium (Figure 6A). As anticipated, *Dlk1* expression was present exclusively in *Dlk1^+/+^* pericardial specimens, but it did not differ between injured and uninjured pericardium as expected from the MI studies (Figure 6B). In agreement with the above in vitro data, *Itgb8* expression was higher in *Dlk1^+/+^* versus *Dlk1^−/−^* pericardium, although its level was also independent of injury (Figure 6B). In contrast, the amount of *Procollagen I* and epicardial fibroblast progenitor transcription factor *Tcf21* mRNAs were decreased in *Dlk1^−/−^* as compared with *Dlk1^+/+^* specifically in the injured pericardium (Figure 6B). This supports in vivo the idea that *Dlk1* sustains the Itgb8/TGFβ‐axis and hereby amplifies collagen production and fibrosis.

Finally, we mimicked the in vivo scenario (Figure 6C) of iCF to myofibroblast conversion after MI^1^, ^37^ where substantial amounts of soluble DLK1 is freely available from the PF and blood as found herein (Figure 1). Soluble DLK1 was absent, as expected, in primary derived adult iCFs, but could be transiently expressed and was not affected by TGFβ (Figure 6D). iCFs overexpressing soluble *Dlk1* increased *Procollagen I* expression upon TGFβ stimulation as expected (Figure 6E). However, this response was specifically inhibited by adding an anti‐DLK1 antibody compared with an isotype matched control, which did not affect iCF myofibroblast conversion and *Procollagen I* production (Figure 6E). Together, these data further confirm that peri‐/epicardial Dlk1 stimulates the EMT response of both EDPCs and iCFs to enhance scarring or its expansion after MI through negatively regulating Itgb8 expression.

## 4 DISCUSSION

**FIGURE 7:**
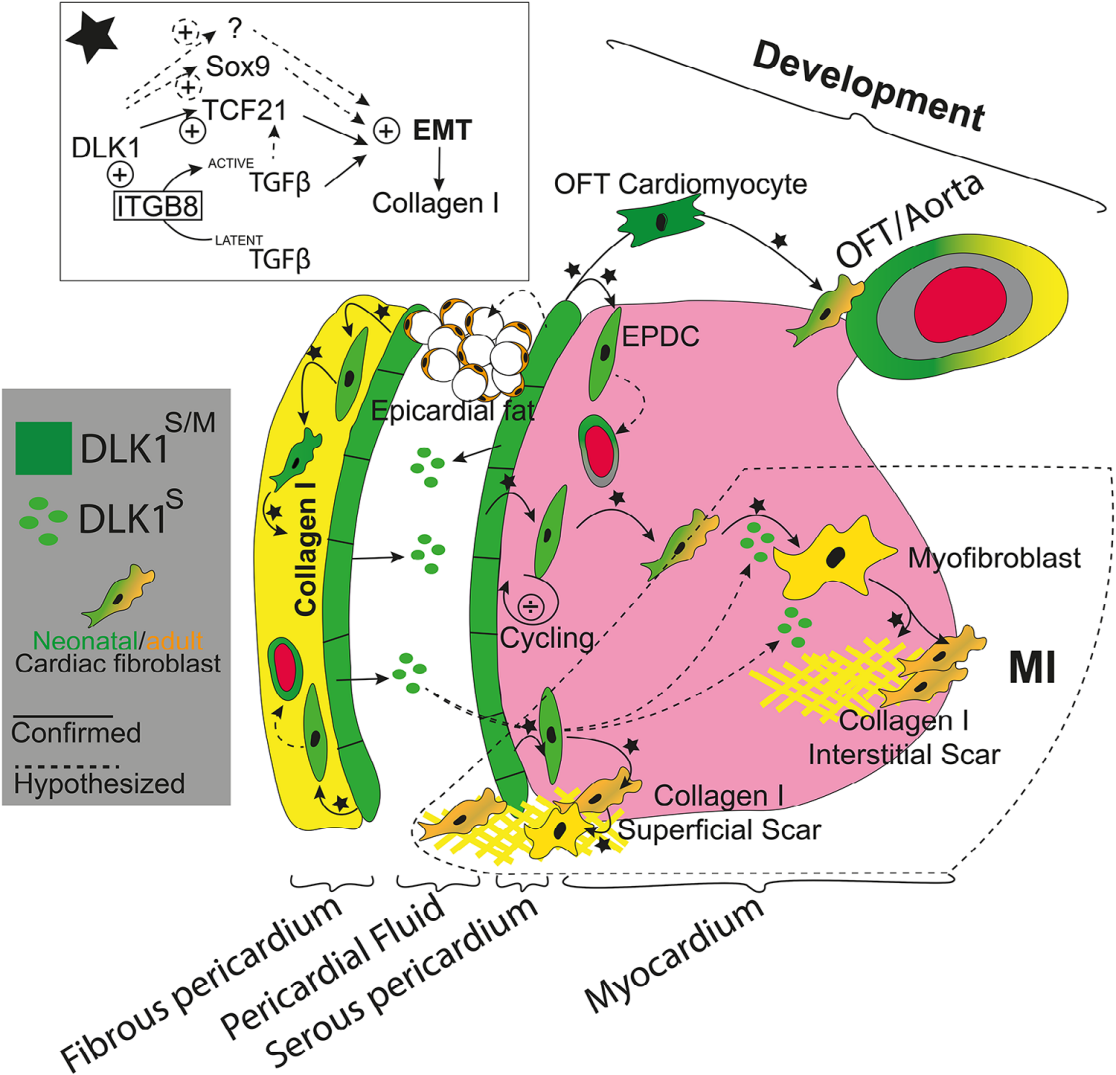
Proposed mechanism of Dlk1 action in the heart during development and disease (MI). In heart development DLK1 (Soluble (DLK1^S^) and membrane (DLK1^M^)) is expressed in all mesothelial cells lining the pericardial space as well as in delaminating EPDCs in the subepicardial space, in developing vasculature, in OFT cardiomyocytes, and in neonatal (pre)fibroblasts. In adulthood, DLK1 is restricted to mesothelial cells (some are negative‐not shown) and the majority of cells in the fibrous pericardium, but also exist in its soluble form shedded into the pericardial fluid. During both development and disease (MI), DLK1 inhibits EPDC proliferation while mediating EMT of EPDCs including their maturation into fibroblasts and myofibroblasts, and in the end, this increase collagen I expression and scar formation after MI (encircled by a dotted line—‐). The underlying mechanism of DLK1 in these EMT scenarios (marked by a star) may be complex and vary but seems to include Itgb8/ TGFβ regulation, Tcf21 enhancement and eventually also regulation of the previously shown Dlk1 target Sox9 as well as other factors.

Emerging knowledge implies that the pericardium plays an important role in the heart remodelling during the development and after MI.^37^ Here, we demonstrate that *Dlk1* is expressed in the EPDC lineage, where it seems to promote EMT with an impact on both heart development and remodelling after MI (Figure 7). Recently, human induced pluripotent epicardial‐derived cell lineages were shown to be marked by DLK1.^41^, ^42^ This is also noted during mouse cardiac development using single‐cell RNA sequencing data.^43^ Yet, contradictory to all these studies, Rodriguez et al.^44^ suggests *Dlk1* expression to be present in adult cardiac fibroblasts and cardiomyocytes, where *Dlk1* is considered to inhibit fibroblast to myofibroblast conversion thereby reducing scarring after MI. However, our data indicate that *Dlk1* sustains *Itgb8* expression levels during development in the epicardial cell lineage. Hereby DLK1 could mediate TGFβ activation, which renders the EPDCs more sensitive for EMT. This results in fibroblast differentiation whereafter *Dlk1* is turned off. In adulthood, *Dlk1* expression is restricted to cells in the pericardium, and DLK1 is secreted into the PF. Whether the observed DLK1 expression in the pericardium and its secretion to PF depends on the pathophysiological state remains unknown as we for ethical reasons only assessed DLK1 in PF from cardiothoracic patients undergoing surgery and not from healthy individuals. Since Dlk1 expression in the mouse is increased in injured pericardium, it is likely that pathological conditions in this compartment increase Dlk1 availability and hereby impact the risk and severity of MI. We have previously shown that increased DLK1 amounts associates with increased pericardial fat,^25^ a key risk for cardiovascular disease. Thus, following an MI, one may consider several Dlk1 mechanisms of action (Figure 7): (i) *Dlk1* may work locally through *Itgb8* or another pathway in the pericardium, stimulating EMT of EPDCs, which then participate in forming the superficial scar after MI^1^, ^37^; and/or (ii) *Dlk1* in EPDCs may activate through *Itgb8* or another pathway TGFβ, which then activates in a paracrine fashion iCFs in the myocardium to undergo myofibroblast conversion^1^, ^37^ with collagen secretion and scar formation and/or (iii) secreted DLK1 may itself work in a paracrine manner affecting the process of myofibroblast conversion in the myocardium.^1^, ^37^

The detailed DLK1 expression pattern within the EPDC compartment agrees with previous discrete observations in which *Dlk1* mRNA has been noted within the OFT endocardium, the epicardium, the atrial septum and the mesenchymal cushions during early life.^17^, ^18^, ^19^, ^20^, ^21^, ^22^ It also very much supports the notion of DLK1 marking epicardial cells derived from induced pluripotent stem cells.^41^, ^42^ Although our data do not exclude that DLK1 may exert an effect on the heart vascular compartment, we focused on the role of *Dlk1* in EPDC and cardiac fibroblast EMT processes. In this regard, *Dlk1* seems to promote EMT of EPDCs and cardiac fibroblasts (Figure 7).

This is also in agreement with previous studies in skin, placenta, lung, and adipose tissue showing that *Dlk1* marks fibroblast and/or fibroblast‐like precursors during development.^39^, ^45^, ^46^ In EPDCs, *Dlk1* seems to affect EMT at least partly through regulation of *Itgb8* expression (Figure 7). This may link *Dlk1* to TGFβ signalling and fibrosis, where ITGB8 is a recently identified activator of TGFβ,^40^ a key mediator of EMT, as well as heart fibrosis after injury.^1^, ^37^ Others have shown that after liver injury DLK1 is expressed by some hepatocytes and, through paracrine signalling, activates hepatic stellate cells to undergo myofibroblast conversion with excessive ECM production.^47^ Likewise, DLK1 is expressed at increased levels in airway fibrosis in chronic obstructive asthma, where it facilitates collagen accumulation through integrin α5β1 signalling.^46^ A similar scenario may occur in the heart following MI. The fibrosis‐promoting effect of DLK1 after MI is supported by our three independent transgenic mouse *Dlk1* designs, which demonstrate that excessive DLK1 expression increases scar size expansion/maturation with collagen accumulation, whereas the absence of DLK1 reduces scar size expansion/maturation. Although scar size is clearly affected by Dlk1 changes, the functional consequence of this is less clear. Since equal EFs were observed for all transgenic controls, we consider the PET robust. However, it is likely that the initial infarct size may be different between the LAD series as they were each performed by different operators. Thus, one cannot compare between the different Dlk1 designs. Moreover, scar size measurement was performed 2 weeks after functional heart assessment, during which period scarring may have escalated. Finally, it is also possible that Dlk1‐dependent or ‐independent compensatory mechanisms may have contributed unequally between the strains to preserve heart function at this early stage of observation. In the future, a later timepoint for functional assessment will be valuable. Nonetheless, we did observe that Dlk1 overexpression by itself leads to wall thinning, which emphasises its impact on the heart. In support of Dlk1 impacting scarring, we demonstrate that all our in vitro and in vivo experimental designs with both EPDCs and cardiac fibroblasts show that DLK1 increases collagen expression. Our data in the *Dlk1^−/−^* mouse suggest that Dlk1 regulates Itgb8 expression and hereby enhance heart fibrosis. Itgb8 consists of a single beta‐chain that forms a heterodimer with an alpha subunit, and was first described in 1991^48^ whereafter it has been annotated as a mediator of TGFb activation.^49^, ^50^, ^51^ Since then, it has been associated with embryo implantation,^52^, ^53^ cancer^54^, ^55^ and the vascular system^56^, ^57^ scenarios also related to Dlk1.^39^ Yet, knowledge on Itgb8 in the heart is limited, but interestingly mice lacking Itgb8 and its intracellular adaptor protein die prematurely at E11.5 due to defective OFT development.^58^ These previous reports on Itgb8 support a link between Dlk1 and Itgb8. However, whether inhibition of Itgb8 in vivo may be used independent of Dlk1 to inhibit cardiac fibrosis still remains elusive. Moreover, although our data suggest that Dlk1 negatively regulate the expression of Itgb8, molecular control of EMT in EPDCs is complex.^37^ Thus, the Dlk1‐Itgb8 axis might not be the only mechanism underlying Dlk1's role in the heart (Figure 7). For example, our observation that *Tcf21* expression is dampened in vivo in injured *Dlk1^−/−^* pericardium fits very well with the known role of Tcf21 as a promotor of cardiac fibroblast EMT. This could suggest that *Dlk1* acts upstream of *Tcf21*. Moreover, we cannot exclude that *Sox9*, a known target of *Dlk1* in PDGFα^+^ mesenchymal precursors^59^ and a confirmed mediator of EMT in EPDCs,^37^ is involved as well (Figure 7).

Our data on the MI model shows that the deletion of Dlk1 reduces scar expansion/maturation, while overexpression of Dlk1 increases scar expansion/maturation after MI. These experimental results contradict the findings by Rodriguez et al.^44^ that Dlk1 inhibits myocardial fibrosis by its expression in cardiomyocytes and cardiac fibroblasts. We speculate that these discrepancies between Rodriguez et al. and our study may reflect the different methods exploited, especially the DLK1 antibodies used and the methods of cardiac cell isolation and analysis. Our results are based on extensive in vitro and in vivo data obtained from mice and humans and with the use of previously described highly specific, validated anti‐DLK1 antibodies^9^, ^31^, ^32^ and primer sets for qRT‐PCR. Thus, we provide strong experimental evidence of scar size measurements after MI in several distinct transgenic Dlk1 mouse strains. Although we cannot exclude any biological reason for these discrepancies, we do however note that our data extend and confirm previous notions^17^, ^18^, ^19^, ^20^, ^21^, ^22^ and recent single cell RNA sequencing data,^41^, ^42^, ^43^ that Dlk1 is expressed solely in the peri‐epicardial compartment, and not in adult cardiac fibroblasts and cardiomyocytes as suggested by Rodriguez et al.^44^

Despite *Dlk1* ^−/−^ animals showing an abnormal heart growth and lifetime pattern, our study indicates that the cardiac phenotype of deleting Dlk1 is relatively mild, whereas abnormal high levels of Dlk1 in the heart seems devastating in agreement with human phenotypes.^35^, ^36^ Patients with high levels of Dlk1 either in the pericardium or systemically may thus be at high risk for developing comprehensive fibrosis in the heart after MI or following pericardial reactivation due to heart surgery. We thus conclude that *Dlk1* is a novel player in the pericardium of the heart, which may be targeted to modify scar size expansion/maturation after MI or other fibrosis‐related heart events for example after heart surgery. Future studies addressing patient specific variations of DLK1 and its association to heart disease may be valuable, as is the testing of site‐specific inhibition of DLK1 in the pericardial compartment to avoid potential adverse events systemically. If successful, this approach can be extended to other scenarios exemplified by age‐related fibrosis, TGFβ‐induced pericardial adhesions after heart surgery or EMT processes related to mesotheliomas.^37^, ^60^

## AUTHOR CONTRIBUTIONS

*Collection of data, data analysis and interpretation, manuscript writing and final approval of manuscript*: C. H. J. *Collection of data, data analysis, manuscript editing and final approval of manuscript*: R. H. J., T. E., C. B., D. G. E., S. F., S. T. B., S. H., L. A. L., A. M. R., L. P. R., M. S., J. D. M., M. T., M. B., S. U. and J. L. *Conception and design, collection of data, data analysis and interpretation, manuscript writing, final approval of manuscript and financial support*: D. C. A.

## CONFLICT OF INTEREST STATEMENT

D. C. A. and C. H. J. together with the University of Southern Denmark and the Region of Southern Denmark have obtained a patent (WO2022/268644 A1) based on the data generated within this study. Otherwise, the authors declare no competing financial interests.

## FUNDING INFORMATION

The work was supported by The Region of Southern Denmark (Forskningspulje), The Danish National Research Council (#09‐073648 and Sapere Aude # 8045‐00019B), The Lundbeck Foundation (#R48‐A4785 and #R313‐2019‐573), Novo Nordisk Foundation (#NNF17OC0028764), Lægeforeningen (#2011‐3271/480853‐109), Tømrermester Alfred Andersen og Hustru's Fond, Hertha Christensens Foundation, Eva and Henry Frænkels Foundation and Odense University Hospital Research Funding.

## ETHICS STATEMENT

All data conform with the ethical legislation in Denmark, and ethical approvals can be found in *Materials and Methods* section.

## Supporting information

Supporting information

## Data Availability

All data are available from the corresponding author upon reasonable request.
