# Supplementary material for: Pericardial delta like non‐canonical NOTCH ligand 1 (Dlk1) augments fibrosis in the heart through epithelial to mesenchymal transition
Source: Clin Transl Med. 2024 Feb 8;14(2):e1565. doi: 10.1002/ctm2.1565 (PMC10851088; doi:10.1002/ctm2.1565)
Supplement: Supplementary file 1 — Supporting information [file CTM2-14-e1565-s001.docx]

**Supplemental Materials**

**Pericardial delta like non-canonical NOTCH ligand 1 (Dlk1) augments fibrosis in the heart through epithelial to mesenchymal transition.**

^1,2^Charlotte Harken Jensen, ^1,2^Rikke H. Johnsen, ^1,3^Tilde Eskildsen, ^4^Christina Baun, ^1,2^Ditte Gry Ellman, ^1,2^Shu Fang, ^1,2^Sara Thornby Bak, ^4^Svend Hvidsten, ^5^Lars Allan Larsen, ^6^Ann Mari Rosager, ^2,7^Lars Peter Riber, ^1,2,3^Mikael Schneider, ^3^Jo De Mey, ^2,8^Mads Thomassen, ^2,8^Mark Burton, ^9^Shizuka Uchida, ^10^Jorge Laborda, ^1,2,3#^Ditte Caroline Andersen

^1^Andersen group, Department of Clinical Biochemistry and Pharmacology (Odense University Hospital), Winsloewparken 25^1st^, DK-5000 Odense, Denmark; ^2^Clinical Institute/University of Southern Denmark, DK-5000 Odense, Denmark; ^3^Cardiovascular and Renal Research/Institute of Molecular Medicine/University of Southern Denmark; ^4^Department of Nuclear Medicine, Odense University Hospital; ^5^Department of Cellular and Molecular Medicine, University of Copenhagen, DK-2200 Copenhagen, Denmark; ^6^Department of Clinical Pathology, Sydvestjysk Hospital, DK-6700 Esbjerg, Denmark; ^7^Department of Cardiothoracic and Vascular Surgery, Odense University Hospital, DK-5000; ^8^Department of Clinical Genetics, Odense University Hospital. ^9^Center for RNA Medicine, Department of Clinical Medicine, Aalborg University, DK-2450 Copenhagen SV, Denmark, ^10^Department of Inorganic and Organic Chemistry and Biochemistry, University of Castilla-La Mancha Medical School, Albacete, Spain.

^#^Correspondence: Ditte C. Andersen; Department of Clinical Biochemistry and Pharmacology; Odense University Hospital; Sdr. Boulevard 29; Odense C; 5000; Denmark.

E-mail: dandersen@health.sdu.dk; phone no.: +45 6550 3975, fax no.: +45 6541 1911.

**Funding Sources**: The work was supported by The Region of Southern Denmark (Forskningspulje), The Danish National Research Council (#09-073648 and Sapere Aude # 8045-00019B), The Lundbeck Foundation (#R48-A4785 and #R313-2019-573), Novo Nordisk Foundation (#NNF17OC0028764), Lægeforeningen (#2011-3271/480853-109), Tømrermester Alfred Andersen og Hustru’s Fond, Hertha Christensens Foundation, Eva and Henry Frænkels Foundation, Odense University Hospital Research Funding.

**Supplemental Figures**


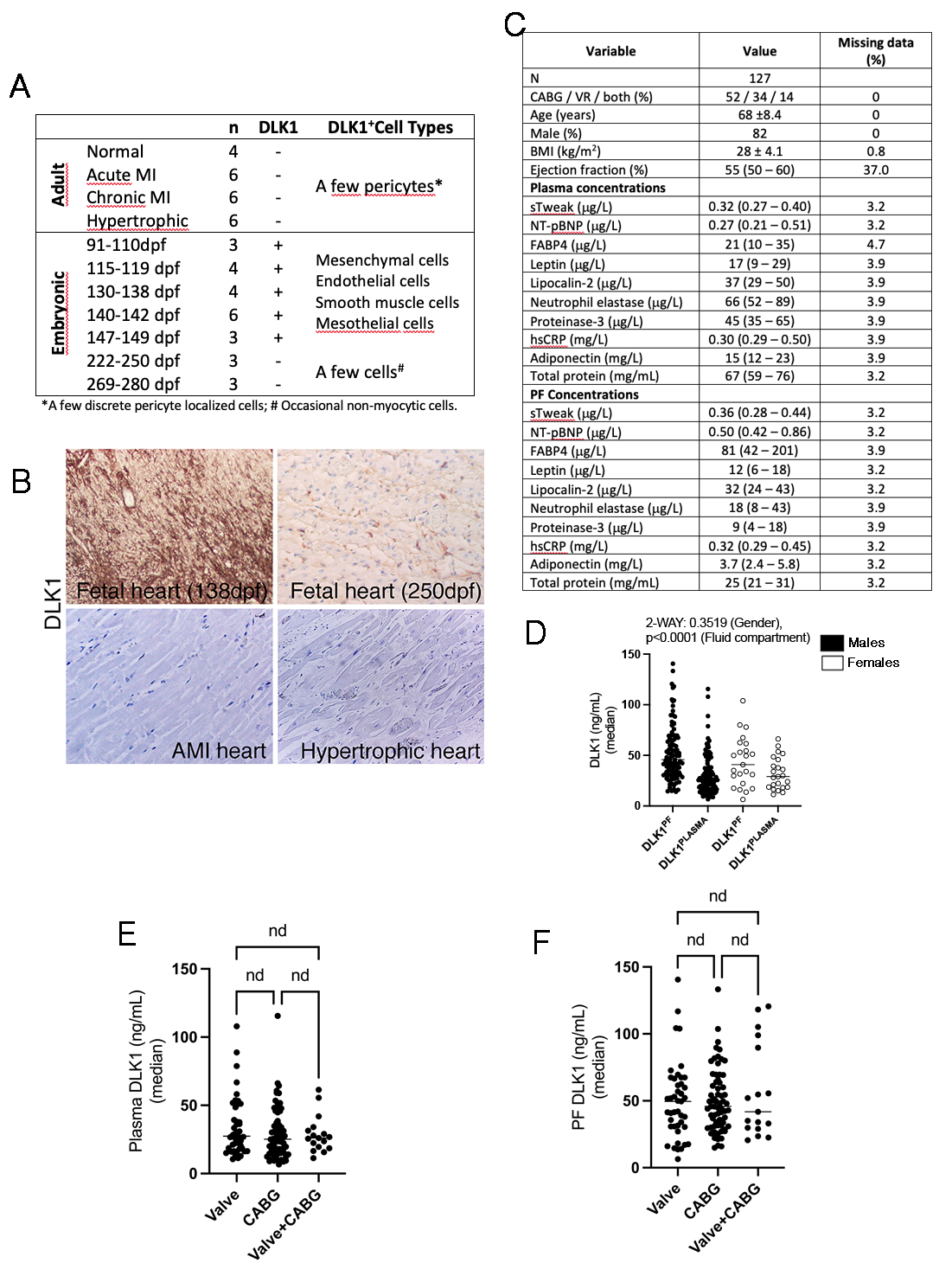


**Supplemental Figure 1.** **Analysis of DLK1 expression in human hearts.** (A) Table summarizing (B) immunohistochemistry results as exemplified with an early (91-149dpf) embryonic heart, a late (222-280dpf) embryonic heart, and adult diseased hearts stained for DLK1. (C) Characteristics of the patient cohort used for plasma and pericardial fluid DLK1 measurements (Main text Figure 1D-E). (D) 2-WAY ANOVA statistical testing confirmed that plasma and pericardial fluid (n=127) DLK1 levels did not depend on the gender of the subjects, and (E-F) Kruskal Wallis 1-WAY ANOVA revealed no association to the etiology of disease (Valve- or artery disease). CABG:Coronary artery bypass surgery.


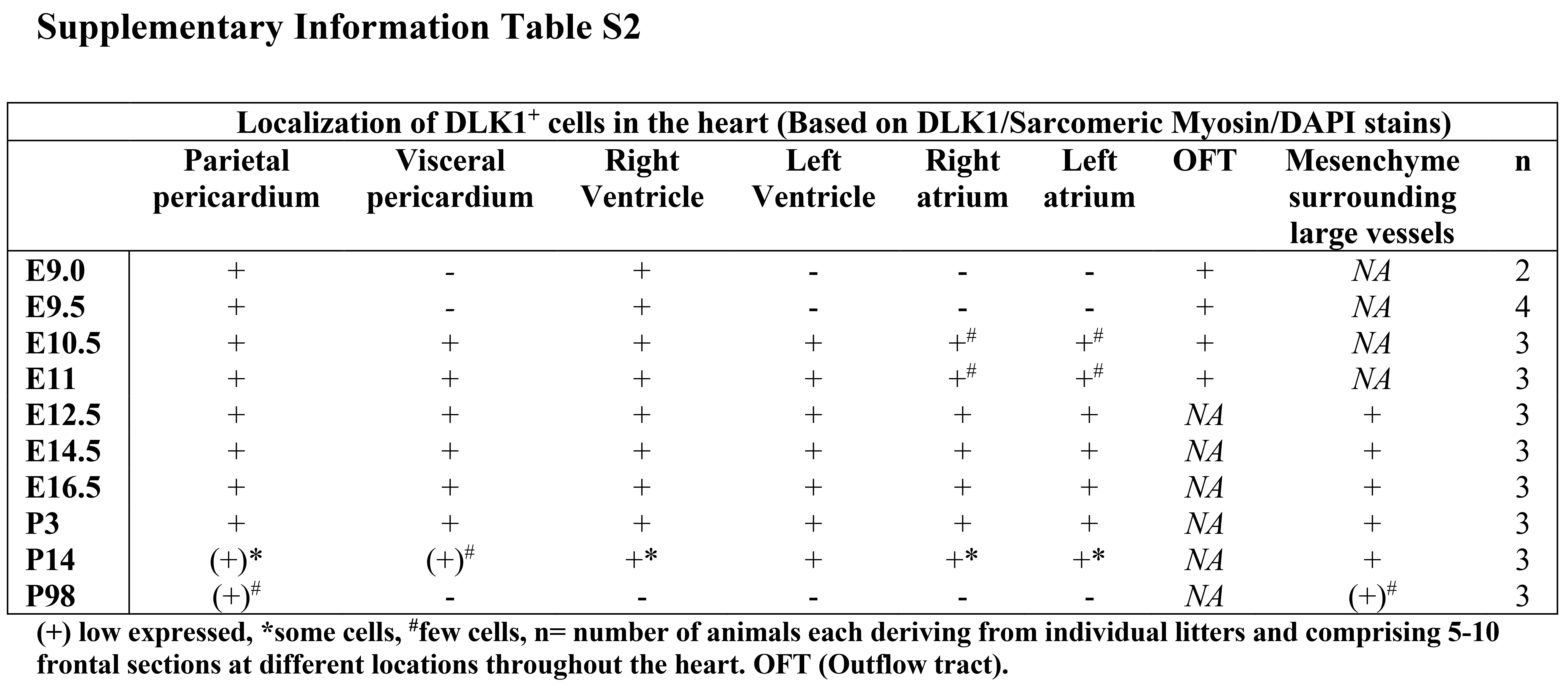


**Supplemental Figure 2.** **DLK1^+^ cell localization in the heart during mouse heart development.**

DLK1/sarcomeric Myosin (MYH1)/Dapi immunofluorescence was performed at different sections throughout the heart and used for assessing localization of DLK1^+^ cells in the heart as it develops. n=2-4 animals deriving from different litters were analyzed for each timepoint. See main Figure 2 for further details.


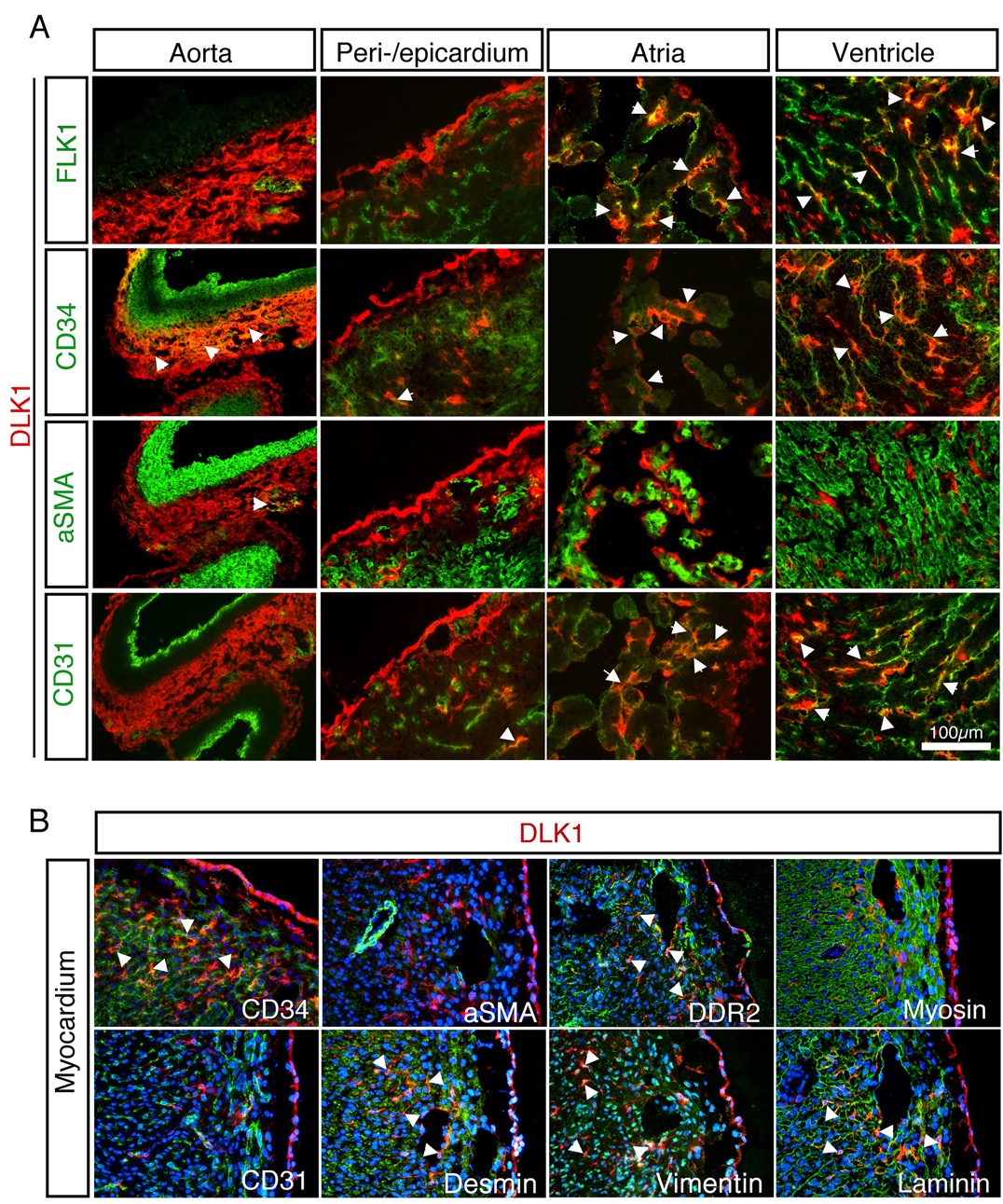


**Supplemental Figure 3.** **Two distinct waves of DLK1^+^ cells appear before- and after birth.** (A) At E16.5 during mouse heart development, DLK1 is co-expressed within the developing ventricles with CD31, FLK1, CD34 suggesting that DLK1 is expressed in endothelial cells, but it is not expressed in endothelial cells in relation to large vessels, but merely in cardiac fibroblasts surrounding the smooth muscle layer in those vessels. (B) After birth at P3, DLK1 is co-expressed within the ventricles with CD34, DDR2, Desmin, Vimentin, and Laminin, but not with aSMA, Myosin, and CD31, which indicate that DLK1 at P3 defines non-myocytes such as immature smooth muscle cells and cardiac fibroblast. n=3 animals deriving from different litters were analyzed, representative pictures are shown and the 100µm scalebar represents all images. DAPI (blue) was used for staining nuclei.

**
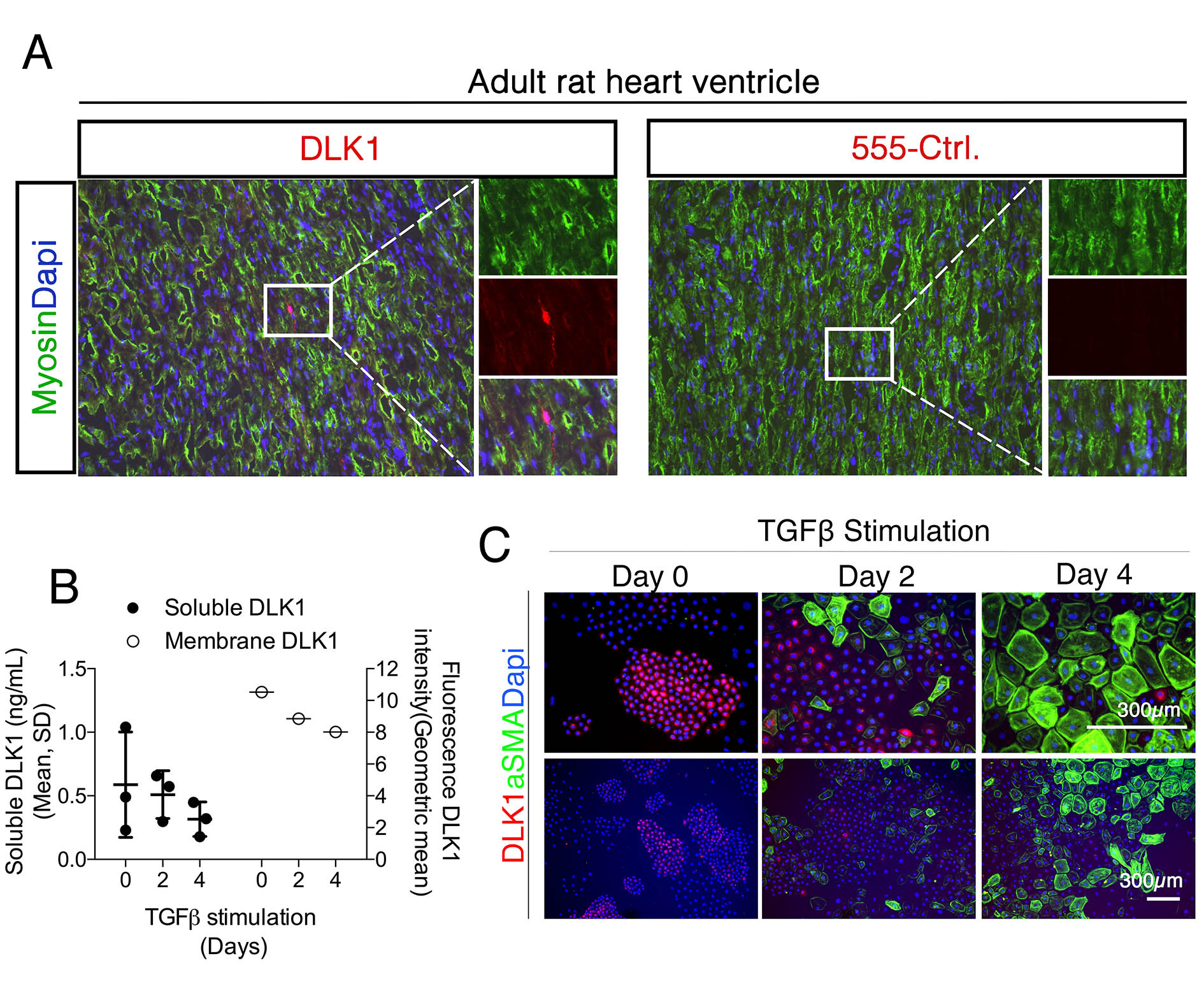
**

**Supplemental Figure 4.** **DLK1 is expressed a few discrete non-myocytes throughout the adult rat myocardium and in cultured rat EPDCs.** (A) Immunofluorescence of adult rat myocardium reveals a few DLK1+ cells with a non-myocyte (Myosin negative) identity. (B-C) Clonal isolation of adult rat EPDCs (n=3) with subsequent expansion and TGFβ stimulation shows that both soluble (ELISA of medium) and membrane bound (Flow cytometry) DLK1 are expressed in rat EPDC as verified by immunofluorescence (C). Yet, DLK1 levels decrease as EPDCs undergo epithelial to mesenchymal transition, forming cardiac fibroblast descendants. (n=3, each comprising 3 replicates).

**
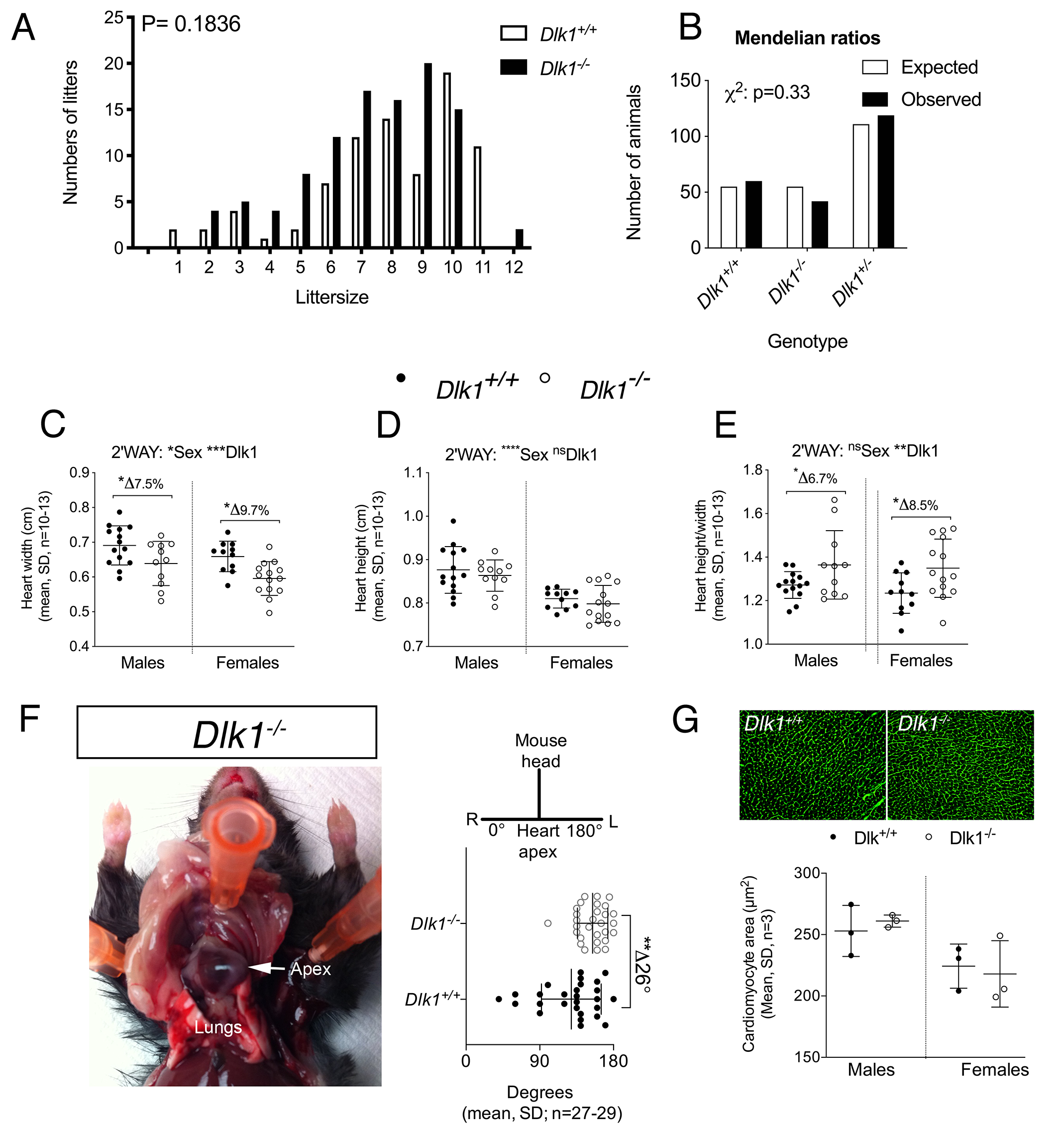
**

**Supplemental Figure 5.** **Characterization of the heart phenotype in *Dlk1* deficient mice.** (A) Three independent breedings of homozygous *Dlk1^+/+^* and *Dlk1^-/-^* were performed and litter sizes were determined to be equal between genotypes. (B) Five breeding pairs heterozygous for *Dlk1* were breeded three times and, the genotype of descendants determined and compared to the expected mendelian ratio. (C-E) The heart width and height as well as the ratio between them were measured in adult *Dlk1^+/+^* and *Dlk1^-/-^* hearts and tested for their dependency on the sex (see Main Figure 4B). (F) Quantification of the heart orientation in the thorax of adult *Dlk1^+/+^* and *Dlk1^-/-^* mice. Data were pooled for female and males since the parameter is independent of sex (Data not shown). (G) Immunofluorescence of adult *Dlk1^+/+^* and *Dlk1^-/-^* hearts (3 females and 3 males for each genotype) using Wheat germ Agglutinin conjugated to Alexa 488 to quantify cardiomyocyte size. For each mouse three non-serial sections in the middle of the heart were analyzed at three different locations (Apex, Mid, Base). Each picture used for analysis comprised around 200 cardiomyocytes that were quantified. Locations were non-significant (Data not shown) and data were therefore pooled for each mouse. For statistical testing we used the non-parametric Wilcoxon matched-pairs signed rank test (A), Chi-square test (B), 2-WAY ANOVA with Two-stage linear step-up procedure of Benjamini, Krieger and Yekutieli posthoc test (C-E, G), and Unpaired t-test (F).

**
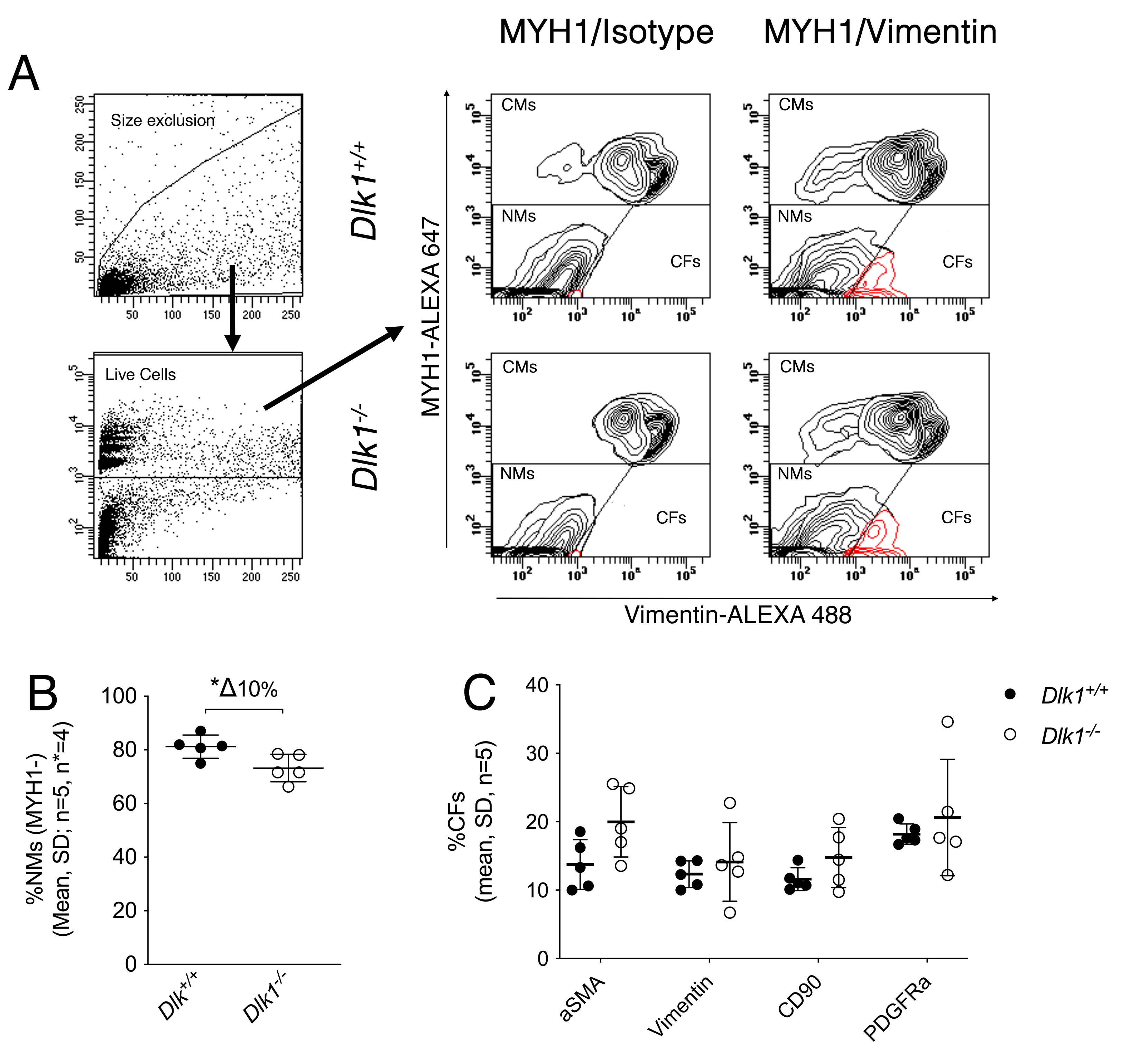
**

**Supplemental Figure 6.** **Percentage of non-myocytes in the heart ventricles of *Dlk1* deficient mice.** Adult *Dlk1+/+* and *Dlk1-/-* ventricles were dissociated and analyzed by flow cytometry. The number of cells analyzed ranged from 17627 to 20141 cells. (A) Representative flow cytometric showing the gating strategy with size exclusion and live cell gating as well as contour plots of MYH1 (Cardiomyocyte marker) and Vimentin (Non-myocyte marker mainly for cardiac fibroblasts). For each antibody used (A-B) to mark non-myocytes, gates were defined by an FMO that included an isotype matched control antibody. (B) For all four non-myocyte markers run (C), the percentage of non-myocytes (MYH1 negative) were quantified and averaged. (C) Quantification of the percentage of cardiac fibroblasts (CFs) using four non-myocyte (mainly fibroblast) markers. For statistical testing we used the non-parametric Mann-Whitney test (B) and a 2-WAY ANOVA with Sidak’s posthoc test (C). *P<0.05

**
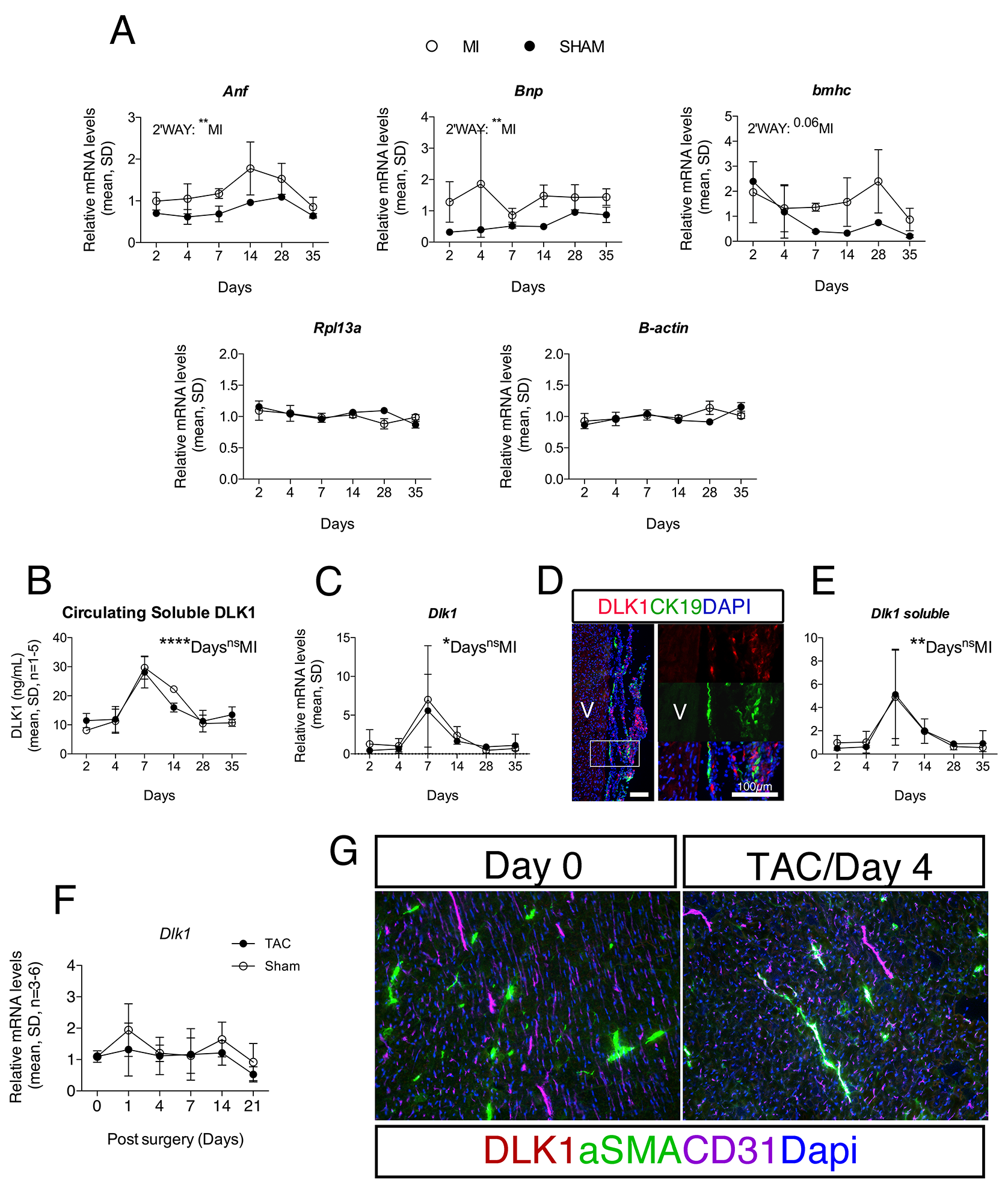
**

**Supplemental Figure 7.** **Myocardial infarction (MI) and Transaortic constriction (TAC) in mice.** (A-E) MI (LAD; n=4-6) or sham (n=1-3) surgery was performed in 10-weeks old female C57bl/6 mice, and hearts were analyzed at different timepoints by normalized relative quantitative RT-PCR for cardiac stress markers (*Anf, Bnp, Bmhc (Myh7)*) and (C, E) *Dlk1* and *soluble Dlk1* transcripts. (B) Soluble DLK1 and (D) DLK1 protein was assessed by ELISA of blood and in hearts sections of ventricle and pericardial tissue (box). Dlk1 was induced by pericardial lesion, but not MI itself. (F) Relative quantitative RT-PCR at different timepoints of *Dlk1* in TAC and Sham hearts from adult mice reveal minimal changes in Dlk1 expression with time and surgery, whereas (G) immunofluorescence substantiate that DLK1+ cells are scarce in number in the heart despite of TAC injury. For both MI and TAC series, transcript levels were normalized against *Rpl13a* and *B-actin* that were stably expressed in both MI and TAC. For statistical testing we used 2-WAY ANOVAs to test for the effect of MI or TAC, respectively. **P<0.01.

| 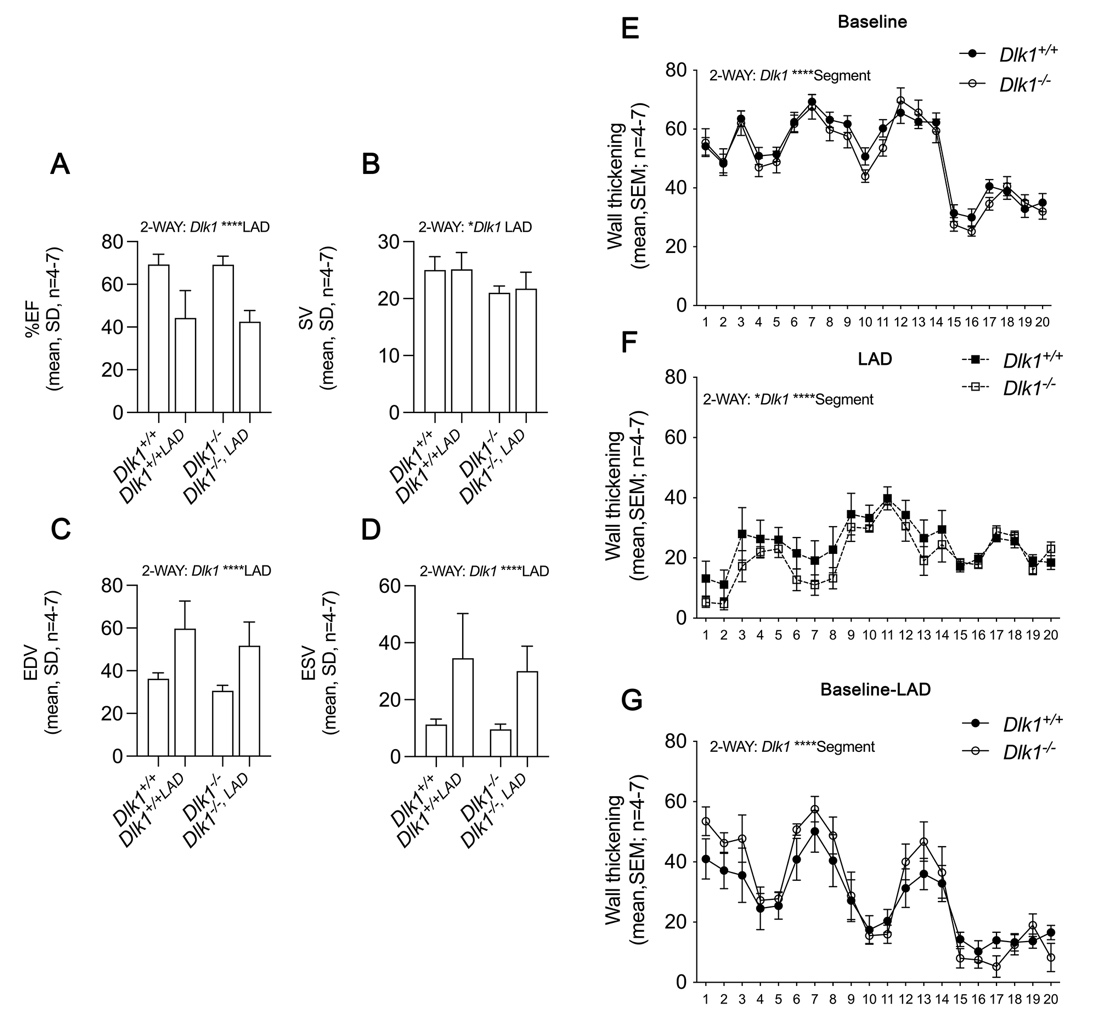 |
| --- |
| **Supplemental Figure 8.** **Positron emission tomography (PET) of *Dlk1^+/+^* and *Dlk1^-/-^* (dlk1 knockout) mice** (10 weeks old) was performed at baseline or 6 weeks after MI surgery (LAD). (A-D) Cardiac performance (%EF, SV, EDV, ESV) was assessed and tested using 2-WAY repeated measures ANOVA. (**E-F**) Wall thickening was quantified for each segment of the heart and tested using ordinary 2-WAY ANOVA for statistical testing. |

| 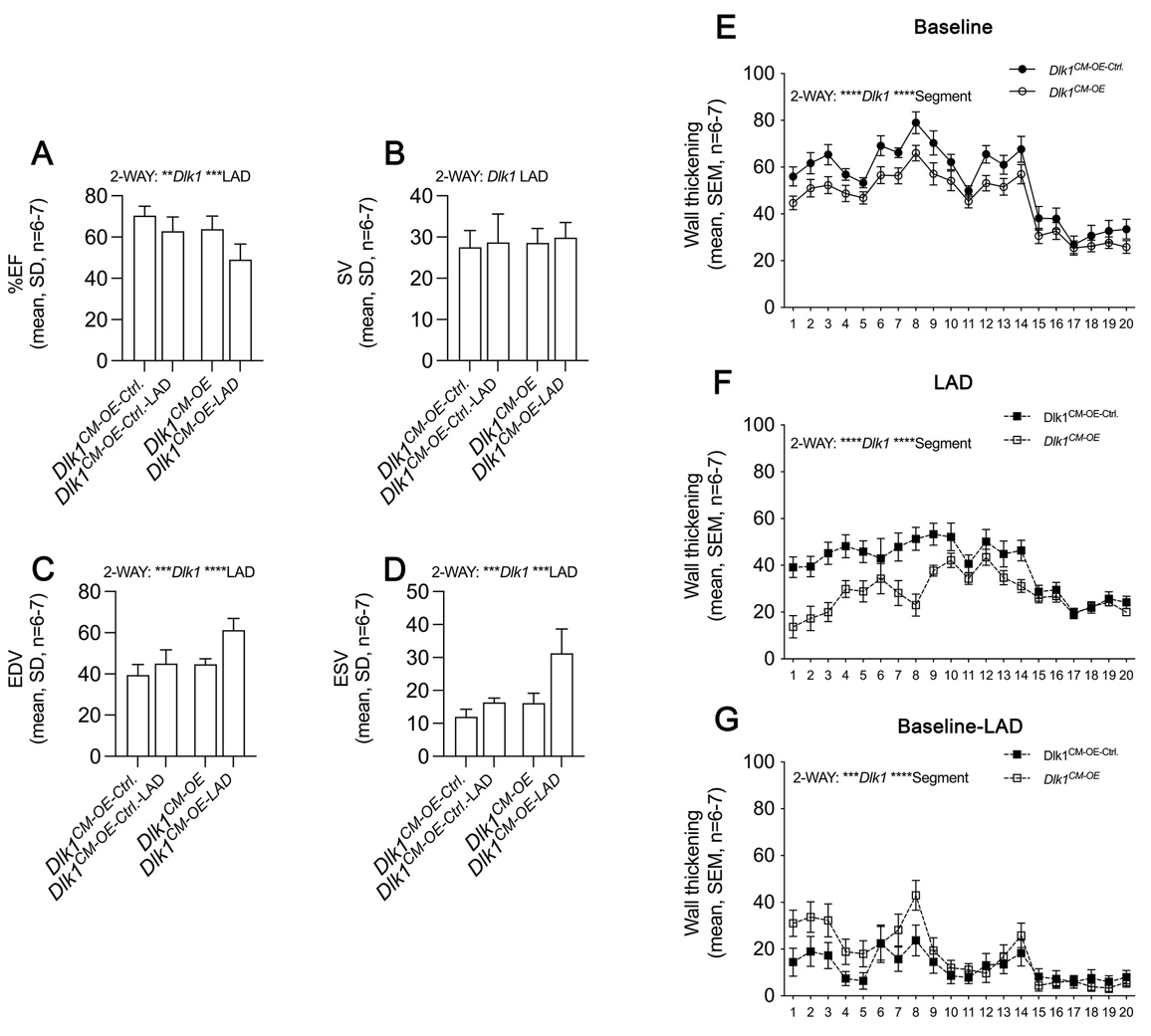 |
| --- |
| **Supplemental Figure 9.** **Positron emission tomography (PET) of *Dlk1^fl/fl^*xαMHC^Cre/+Tam^ (*Dlk1* overexpression in EPDC environment) and their corresponding control mice** (10 weeks old) at baseline or 6 weeks after MI surgery (LAD). (A-D) Cardiac performance (% EF, SV, EDV, ESV) was assessed and tested using 2-WAY repeated measures ANOVA. (E-F) Wall thickening was quantified for each segment of the heart and tested using ordinary 2-WAY ANOVA for statistical testing. |

| 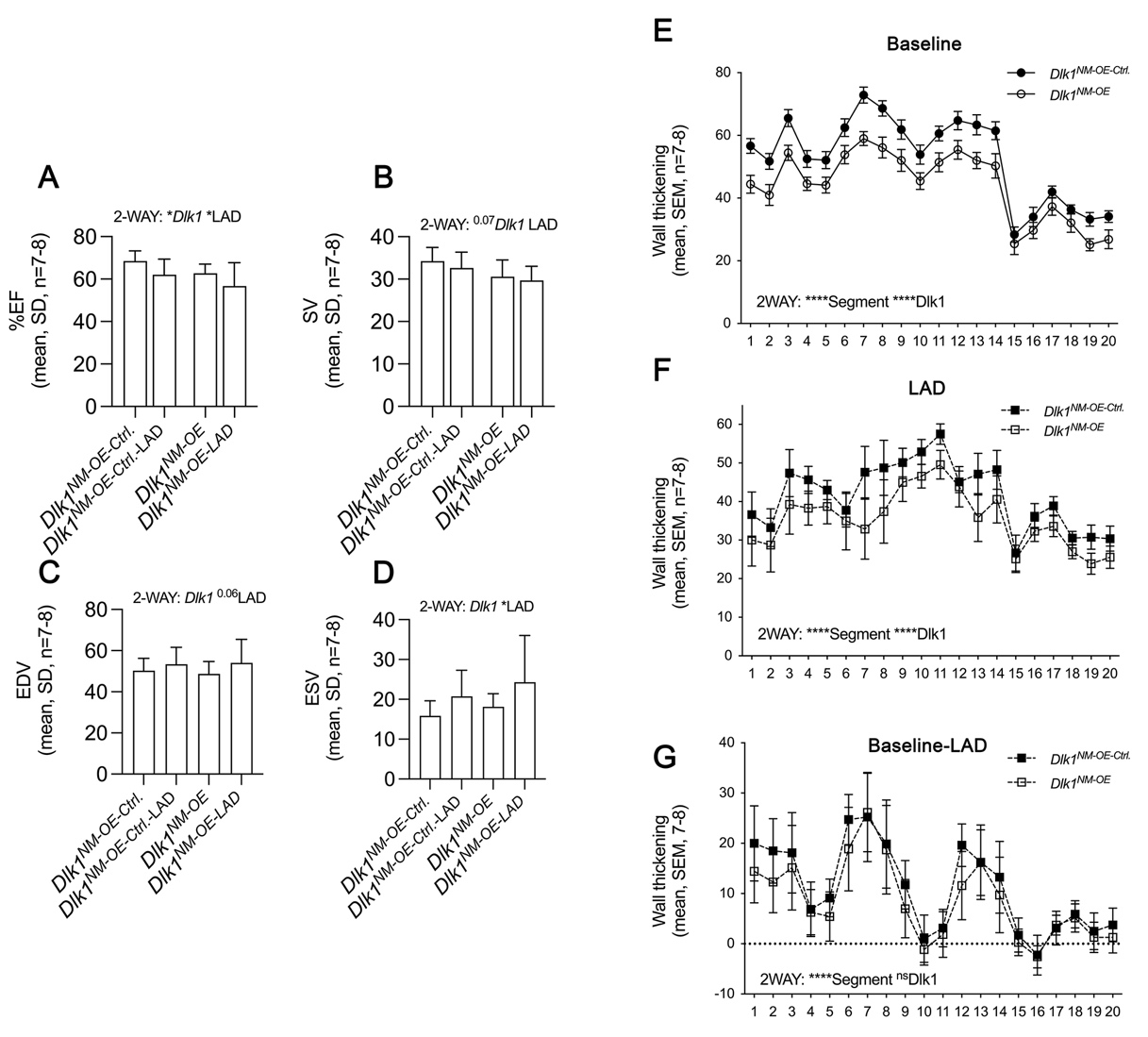 |
| --- |
| **Supplemental Figure 10.** **Positron emission tomography (PET) of *Dlk1^fl/fl^xWT1^GFPCre^* (EPDC lineage *Dlk1* overexpression) and their corresponding control mi**ce (10 weeks old) at baseline or 6 weeks after MI surgery (LAD). (A-D) Cardiac performance (% EF, SV, EDV, ESV) was assessed and tested using 2-WAY repeated measures ANOVA. (E-F) Wall thickening was quantified for each segment of the heart and tested using ordinary 2-WAY ANOVA for statistical testing. |

**
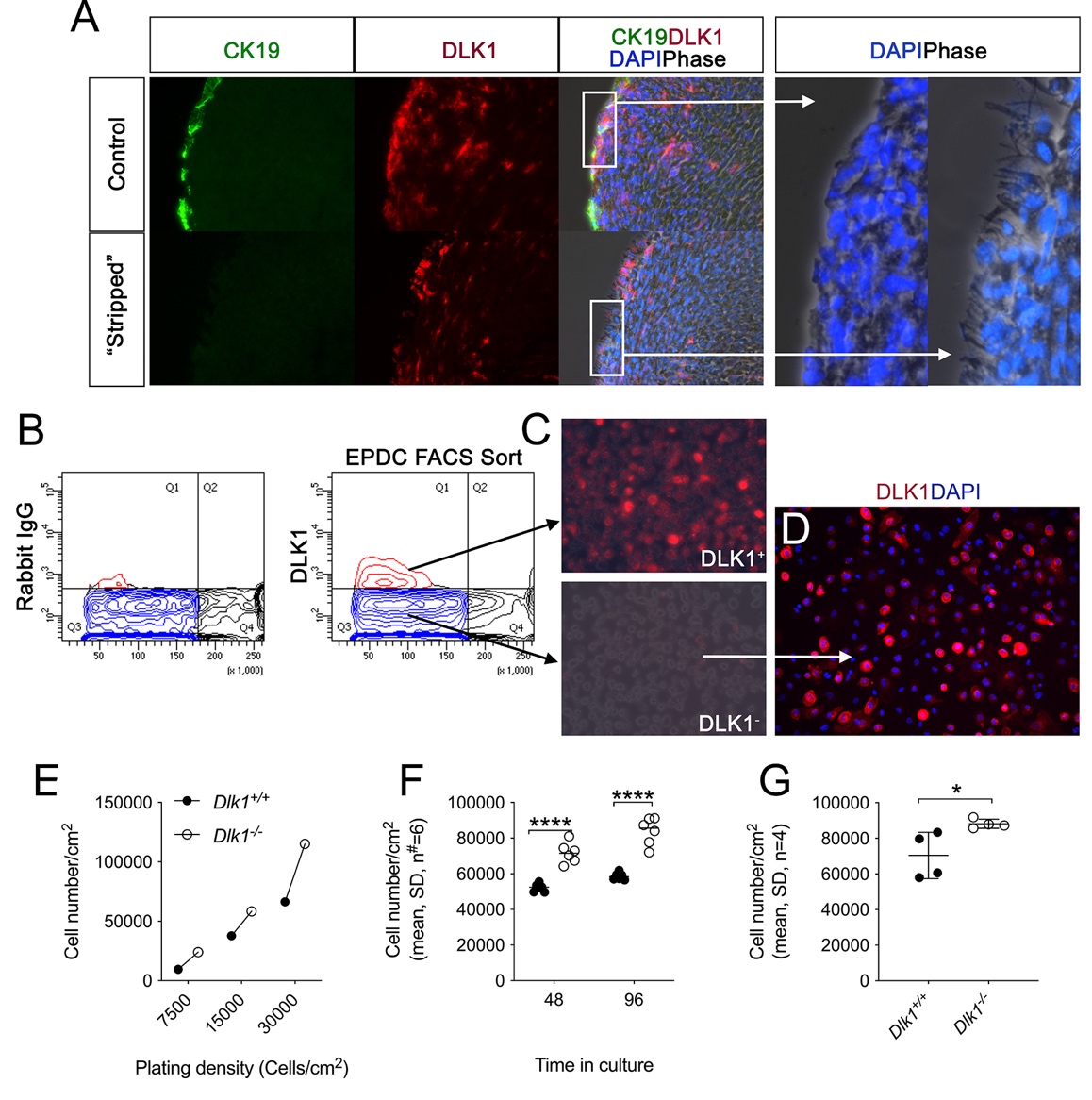
**

**Supplemental Figure 11.** **Isolation and characterization of mouse EPDCs.** (A) Immunofluorescence microscopy of DLK1, Cytokeratin19 (CK19), and Dapi in wildtype mouse hearts before (control) and after EPDC isolation (“Stripped”). (B) Freshly isolated mouse EPDCs were then FACS sorted into (C) DLK1^+^ (Top) and DLK1^-^ (bottom) fractions and (D) cultured for 10 days before cells were analyzed by immunofluorescence microscopy for DLK1 and Dapi. (E-G) Mouse *Dlk1^+/+^* and *Dlk1^-/-^* EPDCs in passage 2 were plated and their proliferation capacity tested in independent series of experiments versus (E) cell plating density after 48hours, where paired data are connected via lines, (F) intraexperiment variation (n^#^=6) after 48- and 96 hours (15.000 cells/cm^2^), and finally (G) after plating 15.000 cells/cm^2^ and culturing for 48hours (n=4). For statistical testing we used a 2-WAY ANOVA with Sidak’s posthoc test (F) and non-parametric Mann-Whitney test (G) with *P<0.05 and ****P<0.0001


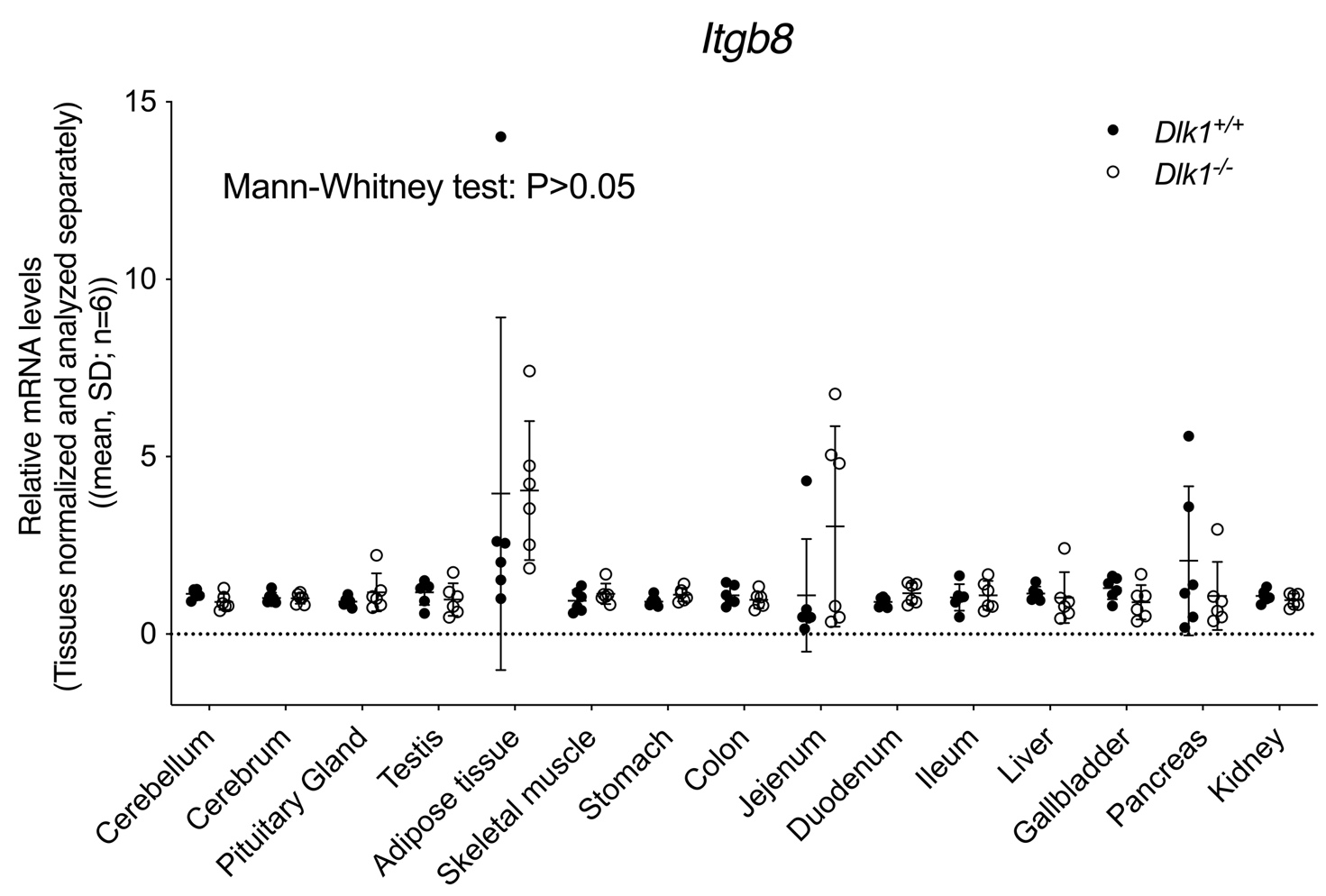


**Supplemental Figure 12.** ***Itgb8* is overall equal between wildtype and Dlk1 deficient mice.** Relative quantitative RT-PCR of *Itgb8* in different tissues from adult *Dlk1^+/+^* and *Dlk1^-/-^* mice (n=6). Transcript levels were normalized and analyzed separately for each tissue according to the qBase platform with several stably expressed endogenous controls. For statistical testing between genotypes, we used non-parametric Mann-Whitney tests.

## Detailed Materials and Methods

See also main text for details.

## Human samples

For DLK1 analysis in the myocardium, we collected i) postmortem heart ventricle tissues from 22 individuals (acute MI- (n=6), chronic MI- (n=6), hypertrophic- (n=6), and normal (n=4) hearts), ii) human fetal heart tissue obtained from abortions (n= 36; 42-280 days post fertilization (dpf)), and iii) biopsies of left ventricle (n=4) from patients undergoing valve replacement surgery. For DLK1 analysis of pericardial specimens, we obtained plasma and pericardial fluids (n=127), as well as parietal pericardial tissue biopsies (n=12) from patients (Supplementary material online, Figure S1C) elected for coronary artery bypass grafting (CABG) or cardiac valve replacement surgeries[6, 20]. We obtained informed consent from enrolled subjects in agreement with ethical legislation. The three human protocols of the study were in accordance with the Declaration of Helsinki and were approved by the Medical Ethical Committee of the Region of Southern Denmark (Protocols: #S-20120065, #S-20180056, #S-20100044).

## Animals and animal samples

Except otherwise indicated, C57BL/6J mice (Taconic, Ejby, DK) were used at the indicated developmental state. Plug breeding was checked in the morning and the evening, and 3-4 different litters at the given timepoints were used in all experiments. *Dlk1^-/-^* mice[10, 16] show absence of *Dlk1* mRNA and protein expression and were maintained by homozygous breeding. The αMHC-MerCreMer strain (*A1cf^(Myh6-cre)1Jmk/J^*, The Jackson Laboratory, Bar Harbor, ME, USA) expresses inducible Cre in cardiomyocytes upon tamoxifen treatment, and it is maintained by homozygous breeding[19]. The WT1^GFPCre^ mouse (WT1^tml(EGFP/cre)Wtp^/J, JAX stock #010911, The Jackson Laboratory, Bar Harbor, ME, USA) expresses Cre in *Wt1* (Wilms tumor 1 homolog) positive secondary heart field precursors[21], and it is maintained by heterozygous breeding to C57bl/6 mice. The *Dlk1^fl/fl^* (Dlk1^flox/flox^) Jl mouse was generated herein by a transgenic insert on the Rosa26-locus and consists of a CAG-promoter and full-length *Dlk1* separated by a stop-sequence flanked by two loxP sites (data not shown). *Dlk1^fl/fl^* x WT1^GFPCre^ mice express *Dlk1* in epicardial cells and descendants hereof such as fibroblasts and smooth muscle cells, while *Dlk1^fl/fl^* x αMHC-MerCreMer express *Dlk1* in cardiomyocytes (EPDC Environment) in an inducible manner. Transgenics were backcrossed to C57BL/6J every third generation to avoid accumulation of non-specific traits. Tail or ear DNA was isolated using a DNeasy kit (Qiagen) and genotype analysis was performed by PCR amplification using the following primers: 5’Dlk1_F: CCAAATTGTCTATAGTCTCCCTC; 5’Dlk1_R: CTGTATGAAGAGGACCAAGG; 5’Neo_F: TTGAACAAGATGGATTGCACGCAGG; 5’Neo_R: GGCTGGCGCGAGCCCCTGATGCTCT, and a Taq DNA polymerase (Invitrogen). Generic Cre Melt Curve Analysis: Cre_F: GCG GTC TGG CAG TAA AAA CTA TC; Cre _R: GTG AAA CAG CAT TGC TGT CAC TT. Internal positive control_F: CTA GGC CAC AGA ATT GAA AGA TCT; Internal positive control_R: GTA GGT GGA AAT TCT AGC ATC ATC C. WT1^GFPCre,TG^; F: ATC GCA GGA GCG GAG AAC; WT1^GFPCre, TG^_R: GAA CTT CAG GGT CAG CTT GC. WT1^GFPCre,Wildtype^_F: CCT ACC ATC CGC AAC CAA G. WT1^GFPCre, Wildtype^_R: CCC TGT CCG CTA CTT TCA GA.

Tamoxifen (Sigma T5648, St. Louis, MO, USA) was dissolved in corn oil (Sigma C8267) at a concentration of 6 mg/mL and administered as i.p. injections at a dose of 40 mg/kg per day for 5 consecutive days. To check for potential non-specific tamoxifen-mediated cardiac effects[12], we administered tamoxifen 4 weeks prior to experiments, and confirmed at baseline that cardiac stress markers and heart function were unaffected (data not shown). Control animals received corn oil in equivalent volumes. TAC left ventricle heart samples were obtained from a previously published [7] series of adult mice (12-weeks, female) that have undergone transverse aortic constriction or sham surgery. At the given timepoints, hearts were harvested, and the dissected left ventricle used for analysis by relative quantitative real-time PCR (qRT-PCR) and immunofluorescence assays. For Dlk1 assessment in rats, we used adult Sprague Dawley rat hearts (n=3) for immunofluorescence. All animal experiments were approved by the Danish Council for Supervision with Experimental Animals (#2016-15-0201-00941).

## Left anterior descending artery (LAD) ligation

Mice were anaesthetized using 4% isoflurane (Baxter) and endotracheally intubated using the BioLite system (Braintree Scientific, Braintree, MA, US). Anesthesia and respiration were maintained by assisted ventilation with a mixture of 2.5% isoflurane and 100% oxygen using a Minivent type 845 mouse ventilator (Hugo Sachs Elektronik Harvard Apparatus, March-Hugstetten, Germany). The heart was accessed by parting of the lower ribs. Permanent ligation of the of the left anterior descending (LAD) coronary artery was performed by placement of a single 8-0 prolene suture (Ethicon, Johnson&Johnson AB, Birkerød, DK) at the surrounding myocardium. Ligation of the LAD coronary artery was visually confirmed by paling of the myocardium distal to the suture. Sham mice underwent the exact same procedure without tightening the suture and removing it completely before closure. The thoracic wall and skin were sutured, and the thoracic cavity was drained using a 23 G venflon. Body temperature was kept between 36-37°C avoiding hypothermia. Temgesic (RB Pharmaceuticals Limited, Slough, Berkshire, UK) was used for pain management. Mice that died immediately after surgery were not included in subsequent analyses. For *heart orientation in thorax,* mice were killed by cervical dislocation, placed on the back, and all four legs fixed. The thorax was gently exposed without disturbing the heart, and the angle of the heart as compared to the head-tail orientation was determined manually using a simple angle goniometer. For *heart to body ratio***,** the value of the heart weight in mg divided by the body weight in g was calculated. For neonatal mice, we determined the heart to body ratio using an average of all hearts and mice weights in a given litter. Finally, for *the heart shape,* the overall shape and macroscopical features of the heart were assessed at dissection and evidenced by representative pictures.

## Pericardial sac lesion (PSL)

Mice were treated as described above for LAD ligation, though, after rib cage opening, the pericardial sac was carefully cut open, but otherwise left undisturbed before closure of muscle- and skin as above.

## F-18-fluorodeoxyglucose positron emission tomography (^18^FDG-PET)

To assess cardiac function, animals underwent ^18^FDG-PET imaging. All animals were anesthetized with a mixture of 1.5-2% isoflurane and 100% oxygen and injected via a tail vein catheter with a bolus of FDG (30.5 ± 3.5 MBq), kept in anesthesia for 15 minutes to prevent muscle uptake, and hereafter returned to their cage. The animals were active only 2 min. after this mild anesthesia. During the handling procedures and the complete FDG uptake period, animals were kept warm by a thermostat-controlled heating pad. Animals were re-anesthetized and placed in a supine position on a heated dedicated PET animal bed 25 min. after injection of ^18^FDG. Electrocardiograph needle electrodes were placed left and right at the thorax and one at the left lower abdomen of the animals to collect the cardiac gating signal. During each imaging session, the respiration rate, temperature and cardiac gating was monitored using the BioVet system (M2M Imaging, Cleveland, Ohio, US). ^18^FDG-PET was performed using a small animal PET scanner (INVEON, Siemens pre-clinical solutions, Knoxville, TN, US) covering the entire mouse with an axial view field of 127 mm[14]. Static PET acquisition was performed for 30 min. after an ^18^FDG uptake period of 31 ± 4 min. The list-mode PET data were framed into 12 cardiac gates and reconstructed using an OSEM3D/MAP algorithm (4 OSEM3D and 18 MAP iterations, requested resolution 0.1 mm) using the Siemens INVEON pre-clinical software. ^18^FDG-PET images were analysed by a blinded physicist with high experience in using the QGS software (Cedars-Sinai Medical Center, Los Angeles, CA, USA). This software allows for automatic processing with only alignment of the heart axes as a variable, thus minimizing the bias of the analysis. Accordingly, functional parameters (EF (%), EDV (µL), ESV (µL)) were established, and the stroke volume (EDV-ESV) was calculated. Moreover, wall motion, stress perfusion and thickness were reported by the 20-segmentation model[17, 18].

## Electrocardiogram (ECG)

Three-lead surface ECGs were recorded using subcutaneous needle electrodes on shielded cables. Electrodes were placed at the left and right side of the thorax and at the left lower abdomen. ECGs were recorded using BioVet CT1 system software (M2M Imaging), with a single channel protocol and no gain applied. Mice were anaesthetized with 1.5% isoflurane (Baxter, Deerfield, IL, USA) in 100% oxygen with spontaneous breathing. The animals were placed in a supine position and needle electrodes were connected and adjusted until a stable signal was obtained. After a few minutes of stabilization, the ECG was recorded for 300 sec. A blinded researcher measured ECG interval lengths. QT interval length was not adjusted to heart rate, as this leads to an underestimation when using isoflurane for anaesthesia[4].

## Coronary artery length

Mice received heparin i.p. 30 min. prior to euthanasia. Immediately after euthanasia the coronary arterial bed was perfused with Microfil mv-122 yellow (Flow Tech Inc., Carver, MA, USA), prepared according to the manufacturer’s instructions, and injected retrogradely through the abdominal part of aorta. The mouse was placed in 4% neutral buffered formalin (NBF, Sigma-Aldrich, cat. no. 225249) for 2 hours. Hereafter the hearts were dissected and returned to 4% NBF. Hearts were individually wrapped in gaze moistened with sterile water and then in plastic wrap and placed in the CT scanner on an 8 mm bed (Siemens INVEON multimodality, pre-clinical solutions, Knoxville, TN, USA). A one-bed step and shoot protocol was performed with 720 rotation steps, a settle time of 4,000 ms, and exposure time of 5,500 ms and 10 sumframes, including 100 dark/light calibrations. Exposure settings were 80 kV and 500 µA and a high magnification level with no binning, giving an effective pixel size of 9.2 µm, an axial FOV of 11.7 mm, and an estimated scan time of 42,552 sec. The images were reconstructed using a Hounsfield calibrated filtered back-projection with no down sampling. Arteries were traced on the obtained CT images using the ImageJ/Fiji plugin Simple Neurite Tracer[13] yielding total coronary artery length. The researcher performing the tracing was blinded regarding sex and genotype.

## Histology and immunohistochemistry

Dissected hearts were embedded in Tissue-Tek (Sakura Finetek Europe, Alpena aan den Rijn, NL) and snap-frozen using isopentane (Sigma-Aldrich). For heart development series, hearts were sectioned frontally, and sliced throughout in steps to reveal all parts of the hearts (three different hearts derived from individual litters were examined). For *scar-size quantification*, hearts were cross-sectioned in steps (50 sections each) starting from the apex towards the base until the scar tissue was absent as visualized by hematoxylin-eosin (HE) and Masson’s Trichrome (MT) staining. The amount of scar tissue and viable myocardium was quantified at each step using Adobe Photoshop, where the observer was blinded to the group and genotype. The total scar size was quantified by calculating the area under the curve across all steps, and then used for statistics. For HE stainings, sections were fixed in 4% NBF for 5 min. before staining with Mayers hematoxylin with citric acid (Amplicon, Odense, DK) and eosin 0.2% (Sakura). Sections for MT staining were fixed for 1 hour in 4% NBF and subsequently prepared in Bouins solution (Sigma-Aldrich) overnight. Staining was performed using Weigert`s Iron Hematoxylin and Trichrome stain (Masson) kit (Sigma-Aldrich). For IHC, sections were fixed in 4% NBF (10 min.) and blocked using 2% bovine serum albumin (BSA) in Tris buffered saline (TBS). Primary antibodies were diluted in 1% BSA. Primary antibodies used included rabbit anti-mouse DLK1[3] (1:2,000, in-house), rabbit anti-human DLK1[11](1:500, in-house), mouse anti-mouse DLK1[8] (C5/C11; 5 µg/mL, in-house), mouse anti-MYH1 (Sarcomeric myosin, 1:300, MF20-c, DSHB), rabbit anti-Islet 1(1:750, Ab20670, Abcam), mouse anti-Islet 1 (1:25-50, 39.4D5, DSHB), goat anti-Desmin (1:50, sc-7559, Santa Cruz Biotechnology, Dallas, TX, USA), mouse-anti alpha smooth muscle actin (aSMA, A5228, 1:2-400; Sigma-Aldrich), rabbit anti-Wilms tumor 1 (WT1, ab15249, Abcam, 1:50), rat anti-Flk1 (1:50, 14-5821-81, eBiosciences), rat anti-Pecam1 (CD31, 553370, 1:50, BD Pharmingen), rat anti -CD34 (1:50, 550537, BD Pharmingen), rat anti-E-cadherin (Cdh5, Ab11512, 1:700, BD Biosciences), rat anti mouse PDGFRa (1:50, ab90967, Abcam), goat anti-DDR2 (1:40, sc-7559, Santa Cruz Biotechnology), mouse anti-Vimentin (Vim, V6389, 1:40, Sigma-Aldrich), rat anti-Laminin (1:50, L0663, Sigma-Aldrich), rat-anti-mouse CD45 (1:50, 550539, BD Pharmingen), mouse-anti-TroponinT (1:2-400, T6277, Sigma-Aldrich), Cytokeratin 19 (1:50, TROMA III, DSHB), goat anti-rat CD31 (1:50, sc-1506, Santa Cruz Biotechnology). Secondary antibodies (all 1:200, Molecular Probes, Eugene, OR, USA) used were either conjugated with Alexa-488, Alexa-555 or Alexa-647. Mounting medium contained DAPI (Vectashield, Vector Labs, Burlingname, CA, USA). For Wheat germ agglutinin (WGA) staining, cryosections were fixed and blocked as described for IHC and incubated for 90 min. with WGA Alexa fluor 488-conjugate (W11261, Molecular Probes) diluted in TBS to 10 μg/mL. For paraffin embedded sections, staining was performed as previously described with the use of EnVision+ (K500711-2, DAKO) and carbazole for visualization[2].

Microscopic examinations of fluorescent-stained sections were performed using a Leica DMI4000B Cool Fluo Package instrument equipped with a Leica DFC340 FX Digital Cam and a Leica DFC 300 FX Digital cam (Leica Microsystems, Ballerup, DK). In all experiments, camera settings and picture processing were applied equally to samples and controls. Histological sections were examined using a Leica M80 stereomicroscope with Leica IC80 HD digital cam (Leica Microsystems). Photoshop (versions up to 21.2.2, Adobe systems Inc.) was used for picture processing.

## Cardiomyocyte size quantification

WGA-stained sections (see above) were examined, and images were taken in areas where cardiomyocytes were cross-sectionally oriented. Cardiomyocyte cross-sectional areas were measured using the ImageJ/Fiji WGA macro (version 3.1, CBS, University of Leicester). Cells with an area <50 µm2 or >1,000 µm2 were excluded. Three sections (middle and on each side) were analyzed for each mouse. Three images were taken at different locations (apex, mid and base) in the lateral left ventricle wall of each section. For each location at least 207 cardiomyocytes were used for size quantification.

## Isolation and culturing of EPDCs

Neonatal EPDCs were isolated as described previously[22]. Briefly, neonatal (day 1-3 postnatal) mice from a given litter were sacrificed by decapitation. Hearts were carefully dissected without disturbing the epicardium and pooled for each litter which then corresponded to one biological independent replicate. EPDCs were dissociated by gentle treatment with 0.3% Trypsin/DNase solution followed by centrifugation and red blood cell lysis. Harvested EPDCs were plated on ECM-coated plates (E1270; Sigma-Aldrich) and cultured in DMEM (Dulbecco’s Modified Eagle’s Medium) supplemented with 20% FBS (Fetal Bovine Serum)/1% PS (Penicillin-Streptomycin) (all from Lonza). Purity was checked by flow cytometry as previously described[22]. Total cell numbers were determined using a Beckman Coulter Counter Z2 fitted with a 100 µm aperture or a NucleoCounter® NC-200 (Chemometec, DK). EPDCs were used for experiments at passage 1-3. Rat EPDCs were isolated and cultured as previously described[5]. Transfection with siRNAs (Itgb8 (ID s115991) and Scramble (Ambion, Thermo Fisher Scientific) was performed as previously described[15]. Briefly, EPDCs were plated 48 hours prior to transfection, and starved for 1 hour before being transfected for 4 hours (20 nM siRNAs), following which medium was changed to DMEM/10% FCS±10 ng TGFβ (R&D Systems).

## Fibroblast isolation and culturing

Adult male C57BL/6J mice (Taconic, Denmark) were sacrificed by cervical dislocation. The hearts were carefully removed, cut into smaller cubes, and dissociated using the mouse and rat neonatal heart dissociation kit (Miltenyi Biotec; 4 samples, 2 hearts/sample) according to the manufacturer’s instructions. Following dissociation, cells were washed in DMEM supplemented with 10% FBS and 1% PS and filtered through a 100 µm strainer. Viability and number of viable cells were determined using a Nucleocounter NC-200 (Chemometec, DK). The cardiac cells were cultured in DMEM supplemented with 10% FBS and 1% PS until they reached a confluency level of 80% (96 h), passaged by trypsinization and frozen at passage 2 (P2) until initiation of the experiment. For experiments, cells were thawed and maintained until they were set up at P4 in 12-well plates. The day after seeding, cells were transfected in DMEM (no FBS and PS) with plasmids harboring full-length *Dlk1* (DLK1FL-pLHCX-HA), soluble *Dlk1* (DLK1E-pLHCX-HA) or an empty control plasmid (pLHCX-HA; 1.25 µg/well, adjusted in molar concentration to DLK1FL-pLHCX-HA) using the Lipofectamine 2000 (Invitrogen) transfection reagent, according to the manufacturer’s recommendations. Four hours after transfection, the transfection reagents were removed and culture medium (DMEM supplemented with 10% FBS and 1% PS) containing or not 10 ng/mL TGFβ (R&D Systems) or DLK1 antibody (CC5+CC11; 5 µg/mL of each; generated in house) or its correlating isotype control (10 µg/mL) was added. The experiment was terminated 48 h after transfection and processed for qRT-PCR (see below).

**Langendorff adapted EasyCell isolation of heart cells**

Perfusion buffer (containing: sodium chloride (Sigma, cat. no. S3014), potassium chloride (Sigma, cat. no. P5405), potassium dihydrogen phosphate (Merck, cat. no. 1.048.730.250), sodium dihydrogen phosphate (Merck, cat. no. 1.06586.0500), magnesium sulfate heptahydrate (Sigma, cat. no. 63138) and N-2-hydroxyethylpiperazine-N-2-ethane sulfonic acid (Fisher Scientific, cat. no. 15630-056, sodium bicarbonate (Merck, cat. no. 1.06329.1000), Taurine (Sigma, cat. no. T8691), 2,3-butanedione monoxime (Sigma, cat. no. B0753) and glucose (Sigma, cat. no. G6152)) was loaded on the EasyCell System (Hugo Sachs Elektronik, Harvard Biosciences, cat. no. 803) in the large reservoir, whereas digestion buffer (perfusion buffer containing 2.4 mg/mL collagenase II (Worthington. Cat. no. LS004177)) was loaded in the small reservoir at least 15 minutes before use. Hearts were taken from mice injected with 0.5 mL 100 IU/mL heparin (Sygehusapotek Fyn, cat. no. 741835) in sterile phosphate buffered saline (PBS) 15 minutes prior to sacrifice. Hearts were then placed in 25 mL perfusion buffer before they were cannulated, placed on the EasyCell system, and then digested for 13-16 min. After digestion the heart was cut from the cannula just below the atria in 2.5 mL, 37°C warm, digestion buffer. The ventricles were torn in 10-12 small pieces, whereafter 5 mL of stopping buffer (perfusion buffer containing 10% FBS (Sigma-Aldrich cat. no. 217135) was added and the solution was gently pipetted 50 times. Hereafter the cell suspension was filtered through a 300 µm PluriStrainer (PluriSelect, cat. no. 45-50300-03), centrifuged before the cells were resuspended in 10 mL of stopping buffer, and centrifuged again. The cells were then resuspended 1 % NBF (Sigma-Aldrich, cat. no. 225249) in HBSS (Lonza, cat. no. 10-527F) containing 5% FBS and 1% PS (Lonza, cat. no. DE17-602E) for fixation. Finally, the cells were washed twice in HBSS/5% FBS/1% PS and stored in HBSS/5% FBS/1% containing 0.05% sodium azide (Sigma, cat. no. 26628-22-8) at 4°C until further analysis.

## Relative quantitative real time PCR (qRT-PCR)

Total RNA was extracted from cells or tissues by Trizol (Thermo Fisher Scientific) and for cDNA synthesis 0.3 - 0.4 µg of total RNA was reverse transcribed with High-Capacity cDNA RT kit (#4368813, Thermo Fisher Scientific). Quantitative real-time polymerase chain reactions (qRT-PCR) using customized primer sets (Supplementary Table 1) were performed as previously described[1] and run on a 7900HT Fast Real-time PCR system (Applied Biosystems) or a QUANTSTUDIO 7 FLEX instrument (Thermo Fisher Scientific). Quantitative RT-PCR data were obtained by normalizing the raw data against multiple stably expressed endogenous control genes as determined by the qBase Plus platform [9].

## Flow cytometry and fluorescence-activated cell sorting (FACS)

For flow cytometry, fixed cardiac cells (either EPDCs, cardiac fibroblasts or all heart cells) were permeabilized with PBS containing 1% BSA and 0.1% Triton X-100 and stained with one of the following primary antibodies: aSMA, WT1, Islet1, Vimentin, CD45, CD31, TroponinT, Myh1, CD90, PDGFRa (specified above in the “Histology and Immunohistochemistry” section) for 1 hour in the dark on ice while shaking. After washing, cells were incubated with secondary antibodies conjugated with Alexa 488, Alexa 555 or Alexa 647 (all 1:200, Molecular Probes, Eugene, OR, USA) for 30min in the dark while shaking. For some samples, Hoechst was added for visualizing the DNA. All samples were run at a LSRII flow cytometer (BD Biosciences), and data were analyzed using the FACSDiva software v8.0.1. As previously described[1, 2, 22], we performed fluorescence minus one (FMO) controls, as well as isotype controls for all setups, and excluded cell debris and cell doublets using hierarchical gating. For fluorescence-activated cell sorting (FACS), EPDCs were stained with a Dlk1 antibody or an isotype control, and cells were sorted according to their Dlk1 expression using a FACSAriaIII cell sorter (BD Biosciences).

## mRNA microarray expression profiles

RNA was reverse transcribed using the MessageAmp II Enhanced kit (Applied Biosystems) and hybridized to Affymetrix^®^ GeneChips (Mouse Genome 430 2.0 Array) that were run using the GeneChip Scanner 3000 (Affymetrix, Santa Clara, CA, USA). Data analysis was performed using the open-source R software (Bioconductor); expression indexes were calculated using rma and data was normalized using the quantile method. Differentially gene expression analyses were performed by the Students t-test with a False Discovery Rates (FDR) to adjust for multiple testing. The expression levels of the Dlk1 affected genes were illustrated in a heatmap using the *ComplexHeatmap* R-package.

**ELISA**

For mice, blood was collected in T-MLH (heparinized) tubes (CAPIJECT capillary micro collection tubes, Tokyo, Japan), and plasma was obtained for further analysis. Corresponding human blood and pericardial fluid (PF) samples were collected as previously described[6]. Quantification of human and mouse DLK1 was performed using in-house developed sandwich ELISAs. Briefly, for the human DLK1 ELISA a polyclonal monospecific rabbit anti-human DLK1 antibody was used as catcher antibody, while a biotinylated F(ab)_2_ fragment of the same antibody served as detector antibody[11]. The mouse DLK1 ELISA was based on two monoclonal antibodies, CC5 and CC11 as catcher/detector antibodies respectively[8]. Both assays utilized amniotic fluid (human or mouse as appropriate) as a source of native DLK1 for calibrators and quality controls. Horseradish peroxidase-labelled streptavidin (Invitrogen, Camarillo, CA, USA) was used as conjugate and Ortho-Phenylenediamine (Kem-En-Tec Diagnostics a/s, Taastrup, Denmark) as substrate. Absorbance was measured at 490 nm.

## Statistical analyses

All data are presented in the figures, and each analysis consisted of at least three independent experiments designated n, and when indicated n* refers to the number of animals in each experiment. Statistical significance of the difference between means was determined by either two-tailed *t*-tests, or by one- or two-way ANOVA followed by appropriate post-hoc tests as indicated. The choice of test included normal distribution of the data. Clinical values are presented using the median and interquartile range (IQR). Associations between DLK1 and clinical parameters of interest from a previous cohort[20] were evaluated by correlation analysis using Spearman's rank correlation coefficient. The GraphPad Prism (9.0 Mac version) software was used for all statistical analyses. *P<0.05, **P<0.01, ***P<0.001, ****P<0.0001, ns (not significant).

**Supplemental Table S1 (primer sequences)**

|  | **Forward** | **Reverse** |
| --- | --- | --- |
| *hDLK1* | CTATGGGGCTGAATGCTTCC | CTCTATCACAGACCTCCCCG |
| *hB2M* | TGTGCTCGCGCTACTCTCTC | CTGAATGCTCCACTTTTTCAATTCT |
| *hATP6* | CTGAATGCTCCACTTTTTCAATTCT | CGACAGCGATTTCTAGGATAGTCAG |
| *hCOX4A* | TGAGATGAACAGGGGCTCGAAC | TTCGTAGTCCCACTTGGAGGCTAAG |
| *mDlk* | GAAATAGACGTTCGGGCTTG | AGGGAGAACCATTGATCACG |
| *mDlk1^protease site^* | GCCCGAGCAACACATCCTGAAG | TCCCTCGGTGAGGAGAGGGGT |
| *mGapdh* | GTCGGTGTGAACGGATTTGGC | TGAAGGGGTCGTTGATGGCA |
| *mbeta-actin* | GCTGTATTCCCCTCCATCGTG | CACGGTTGGCCTTAGGGTTCAG |
| *mIgtb8* | GGGATGTGTGTGCTGGGCATGG | GTGCCTCTCCCGCTGCAAACT |
| *mB2m* | ATGGCTCGCTCGGTGACCCT | TTCTCCGGTGGGTGGCGTGA |
| *mProcollagen I* | AAGACGGGAGGGCGAGTGCT | AACGGGTCCCCTTGGGCCTT |
| *mTGF-beta* | GTGGACCGCAACAACGCCAT | CAGCAATGGGGGTTCGGGCA |
| *mTcf21* | CCAGTCAACCTGACTTGGCCC | TCGAGAGCGCAGGGTTCCCA |
| *mAnf* | ATTGACAGGATTGGAGCCCAGAGT | TGACACACCACAAGGGCTTAGGAT |
| *mBnp* | GATGCAGAAGCTGCTGGAGCTGA | TCTGCTGGACCCGGAGGGTG |
| *mMyh7* | GCTGCCCCGGTTTTCAGGTCC | CACGCACTGTCAGCACCTTCTGG |
| *mRpl13a* | CGAAGATGGCGGAGGGGCAG | ACCACCACCTTCCGGCCCAG |

## Supplementary References

1. Andersen DC, Laborda J, Baladron V, Kassem M, Sheikh SP, Jensen CH (2013) Dual role of delta-like 1 homolog (DLK1) in skeletal muscle development and adult muscle regeneration. Development 140:3743-3753 doi:10.1242/dev.095810

2. Andersen DC, Petersson SJ, Jorgensen LH, Bollen P, Jensen PB, Teisner B, Schroeder HD, Jensen CH (2009) Characterization of DLK1+ cells emerging during skeletal muscle remodeling in response to myositis, myopathies, and acute injury. Stem Cells 27:898-908 doi:10.1634/stemcells.2008-0826

3. Bachmann E, Krogh TN, Hojrup P, Skjodt K, Teisner B (1996) Mouse fetal antigen 1 (mFA1), the circulating gene product of mdlk, pref-1 and SCP-1: isolation, characterization and biology. Journal of reproduction and fertility 107:279-285

4. Boukens BJ, Rivaud MR, Rentschler S, Coronel R (2014) Misinterpretation of the mouse ECG: 'musing the waves of Mus musculus'. J Physiol 592:4613-4626 doi:10.1113/jphysiol.2014.279380

5. Bronnum H, Andersen DC, Schneider M, Sandberg MB, Eskildsen T, Nielsen SB, Kalluri R, Sheikh SP (2013) miR-21 promotes fibrogenic epithelial-to-mesenchymal transition of epicardial mesothelial cells involving Programmed Cell Death 4 and Sprouty-1. PLoS One 8:e56280 doi:10.1371/journal.pone.0056280

6. Elie AG, Jensen PS, Nissen KD, Geraets IM, Xu A, Song E, Hansen ML, Irmukhamedov A, Rasmussen LM, Wang Y, De Mey JG (2016) Adipokine Imbalance in the Pericardial Cavity of Cardiac and Vascular Disease Patients. PLoS One 11:e0154693 doi:10.1371/journal.pone.0154693

7. Eskildsen T, Schneider, M., Zha, P., Sandberg, M. B., Almind Knudsen, L., Andersen, D. C., Sadoshima, J., & Sheikh, S. P. (2015) Comprehensive Microarray Analysis Identify Dysregulated MicroRNAs in Pressure Overload Affected Hearts. Translational Biomedicine, <https://findresearcher.sdu.dk:8443/ws/portalfiles/portal/182299619/comprehensive_microarray_analysis_identify_dysregulated_micrornas_in_pressure_overload_affected_hearts.pdf> Special issue.

8. Figeac F, Andersen DC, Nipper Nielsen CA, Ditzel N, Sheikh SP, Skjodt K, Kassem M, Jensen CH, Abdallah BM (2018) Antibody-based inhibition of circulating DLK1 protects from estrogen deficiency-induced bone loss in mice. Bone 110:312-320 doi:10.1016/j.bone.2018.02.030

9. Hellemans J, Mortier G, De Paepe A, Speleman F, Vandesompele J (2007) qBase relative quantification framework and software for management and automated analysis of real-time quantitative PCR data. Genome Biol 8:R19 doi:gb-2007-8-2-r19 [pii]

10.1186/gb-2007-8-2-r19

10. Jensen CH, Kosmina R, Ryden M, Baun C, Hvidsten S, Andersen MS, Christensen LL, Gastaldelli A, Marraccini P, Arner P, Jorgensen CD, Laborda J, Holst JJ, Andersen DC (2019) The imprinted gene Delta like non-canonical notch ligand 1 (Dlk1) associates with obesity and triggers insulin resistance through inhibition of skeletal muscle glucose uptake. EBioMedicine 46:368-380 doi:10.1016/j.ebiom.2019.07.070

11. Jensen CH, Krogh TN, Stoving RK, Holmskov U, Teisner B (1997) Fetal antigen 1 (FA1), a circulating member of the epidermal growth factor (EGF) superfamily: ELISA development, physiology and metabolism in relation to renal function. Clin Chim Acta 268:1-20 doi:10.1016/s0009-8981(97)00152-6

12. Koitabashi N, Bedja D, Zaiman AL, Pinto YM, Zhang M, Gabrielson KL, Takimoto E, Kass DA (2009) Avoidance of transient cardiomyopathy in cardiomyocyte-targeted tamoxifen-induced MerCreMer gene deletion models. Circ Res 105:12-15 doi:10.1161/CIRCRESAHA.109.198416

13. Longair MH, Baker DA, Armstrong JD (2011) Simple Neurite Tracer: open source software for reconstruction, visualization and analysis of neuronal processes. Bioinformatics 27:2453-2454 doi:10.1093/bioinformatics/btr390

14. Magota K, Kubo N, Kuge Y, Nishijima K, Zhao S, Tamaki N (2011) Performance characterization of the Inveon preclinical small-animal PET/SPECT/CT system for multimodality imaging. European journal of nuclear medicine and molecular imaging 38:742-752 doi:10.1007/s00259-010-1683-y

15. Mortensen SB, Jensen CH, Schneider M, Thomassen M, Kruse TA, Laborda J, Sheikh SP, Andersen DC (2012) Membrane-tethered delta-like 1 homolog (DLK1) restricts adipose tissue size by inhibiting preadipocyte proliferation. Diabetes 61:2814-2822 doi:10.2337/db12-0176

16. Raghunandan R, Ruiz-Hidalgo M, Jia Y, Ettinger R, Rudikoff E, Riggins P, Farnsworth R, Tesfaye A, Laborda J, Bauer SR (2008) Dlk1 influences differentiation and function of B lymphocytes. Stem cells and development 17:495-507

17. Schaefer WM, Lipke CS, Nowak B, Kaiser HJ, Buecker A, Krombach GA, Buell U, Kuhl HP (2003) Validation of an evaluation routine for left ventricular volumes, ejection fraction and wall motion from gated cardiac FDG PET: a comparison with cardiac magnetic resonance imaging. European journal of nuclear medicine and molecular imaging 30:545-553 doi:10.1007/s00259-003-1123-3

18. Sharir T, Berman DS, Waechter PB, Areeda J, Kavanagh PB, Gerlach J, Kang X, Germano G (2001) Quantitative analysis of regional motion and thickening by gated myocardial perfusion SPECT: normal heterogeneity and criteria for abnormality. Journal of nuclear medicine : official publication, Society of Nuclear Medicine 42:1630-1638

19. Sohal DS, Nghiem M, Crackower MA, Witt SA, Kimball TR, Tymitz KM, Penninger JM, Molkentin JD (2001) Temporally regulated and tissue-specific gene manipulations in the adult and embryonic heart using a tamoxifen-inducible Cre protein. Circ Res 89:20-25 doi:10.1161/hh1301.092687

20. Yang K, Deng HB, Man AWC, Song E, Zhang J, Luo C, Cheung BMY, Yuen KY, Jensen PS, Irmukhamedov A, Elie A, Vanhoutte PM, Xu A, De Mey JGR, Wang Y (2017) Measuring non-polyaminated lipocalin-2 for cardiometabolic risk assessment. ESC Heart Fail 4:563-575 doi:10.1002/ehf2.12183

21. Zhou B, Ma Q, Rajagopal S, Wu SM, Domian I, Rivera-Feliciano J, Jiang D, von Gise A, Ikeda S, Chien KR, Pu WT (2008) Epicardial progenitors contribute to the cardiomyocyte lineage in the developing heart. Nature 454:109-113 doi:10.1038/nature07060

22. Aagaard KS, Ganesalingam S, Jensen CH, Sheikh SP, Andersen DC (2013) Poor engraftment potential of epicardial progenitors upon intramyocardial transplantation into the neonatal mouse heart. Int J Cardiol 168:4360-4362 doi:10.1016/j.ijcard.2013.05.061
